# Specifics and Methods of Inhibiting the Underfilm Corrosion of Carbon Steel

**DOI:** 10.3390/polym16060780

**Published:** 2024-03-12

**Authors:** Maxim Petrunin, Tatyana Yurasova, Alevtina Rybkina, Liudmila Maksaeva

**Affiliations:** Frumkin Institute of Physical Chemistry and Electrochemistry, Russian Academy of Sciences, Moscow 119071, Russia; mmvp@bk.ru (M.P.); aa_rybkina@mail.ru (A.R.); lmaksaeva@mail.ru (L.M.)

**Keywords:** metal corrosion, polymeric coatings, underfilm corrosion, organosilanes, self-assembled polymeric siloxane nanolayers, corrosion inhibition

## Abstract

The process of metal dissolution under a delaminated insulating polymer coating (underfilm dissolution) has been studied. For this purpose, we used an experimental setup that simulates the process of corrosion of underground metal structures in the presence of through defects in the polymer coating and/or extended areas of peeling of the polymer coating from the metal (loss of adhesion)—subfilm cavities partially or completely filled with electrolyte. In particular, the distribution of the protective current under a peeled polymer coating was studied, and a sharp decrease in the value of the protective current was shown at a distance of 1–3 cm from the edge of the defect with a gap between the metal and the coating of 1–6 mm. The localized nature of metal corrosion under the exfoliated polymeric coating has been demonstrated. The ratio of the areas with accelerated corrosion to the total area of the metal can be 1 to 100. It has been established that there are areas of anodic dissolution of the metal during cathodic polarization of the entire sample with a peeled coating. The activating effect of carbon dioxide and hydrogen sulfide on the corrosion and anodic dissolution of steel under the coating was shown. So, it has been established that the dissolution current flowing from the anodic sections on a surface can increase approximately 10 times in the presence of carbon dioxide and hydrogen sulfide. A synergistic effect of these compounds on the process of localized underfilm corrosion of steel was detected. It has been developed a mechanism for the formation of localized corrosion damage to steel under a delaminated polymeric coating, which can be the nuclei of corrosion cracks upon reaching a certain level of mechanical loads, i.e., stress corrosion cracking (SCC) of carbon steel. Possible manners of inhibiting underfilm dissolution of metals are considered, and a method for pre-treatment of the surface with solutions of organosilanes, which ensures the formation of surface self-assembled polymeric siloxane nanolayers responsible for inhibiting underfilm corrosion of steel, is proposed.

## 1. Introduction

World experience in the operation of underground metal structures, for example, gas pipelines and gas and oil field distribution networks, shows that more than 50–60% of all pipeline failures (accidents) occur due to corrosion [1,2,3]. Typically, corrosion manifests itself either as deep cavities and pitting leading to through rusting of the pipe wall and, as a result, failure of the pipeline, or as the formation of cracks in the wall of a stressed pipe, their growth, and catastrophic destruction of the pipe along an extended pipeline section (stress corrosion cracking (SCC)). Moreover, in some cases, it is believed that the initial stage of the formation of a corrosion-stressed crack is the development of local corrosion damage (pitting) [4]. Moreover, it is believed that in the range of steel corrosion potentials (i.e., in the absence of electrochemical protection (ECP) or in the case of its low efficiency), the occurrence and growth of cracks is initiated either by active anodic dissolution of steel at the crack tip [5], which usually occurs in the course of pitting development, or a decrease in the strength of the metal at the crack tip due to the penetration of electrolytic hydrogen into the metal (hydrogen embrittlement is the limiting case [6]).

Underground metal structures (for example, underground pipelines) are usually protected from corrosion by polymer coatings [7,8]. However, despite the high anticorrosion efficiency of coatings, it is believed [9,10] that the barrier properties of a coating with respect to oxygen and water have little effect on corrosion since the corrosion rate is much smaller than the diffusion rates of the components required for it [11]. Moreover, under field conditions in the presence of soil electrolyte under peeled coating on pipelines, local corrosion defects (pits and cavities) often occur in places where the coatings peel off from the metal (so-called corrugations), which can initiate stress corrosion cracking [4]. Therefore, it can be assumed that under a peeled-off insulation where the metal dissolves (underfilm dissolution/corrosion), conditions can occur that favor the emergence of a stress-corrosion crack. In view of this, studies on the characteristics of underfilm corrosion, identification of the factors that determine the intensity of this process, and development of methods for suppressing underfilm dissolution are relevant.

The interest in studying the processes of underfilm corrosion that appeared in the mid-20th century [9,10,11,12,13] has not decreased to the present day [14,15,16,17,18,19,20,21,22]. However, despite the many years of research into this problem, the mechanism of underfilm corrosion of metals has still not been fully understood [11,12,15,16,17,18,19,20,21,22,23,24,25]. Therefore, a detailed study of the processes occurring on metal under a polymer film in the presence of an electrolyte at the metal-coating interface is a relevant task. Such a study would make it possible to reliably characterize the underfilm dissolution of a metal, examine the features of underfilm corrosion in detail, identify the factors that determine the intensity of the process, map the metal under a coating to localize the metal surface areas most vulnerable to underfilm corrosion, and ultimately develop methods for counteracting underfilm metal corrosion.

The purpose of this work is to study the corrosion of carbon steel under a polymer coating (i.e., underfilm corrosion of metals), estimate the effect of corrosive environment components, namely, sulfide and carbonate-containing compounds, on corrosion, and analyze the prospects for inhibiting the underfilm corrosion of such common structural metals as carbon steel and aluminum.

## 2. Materials and Methods

**Figure 1:**
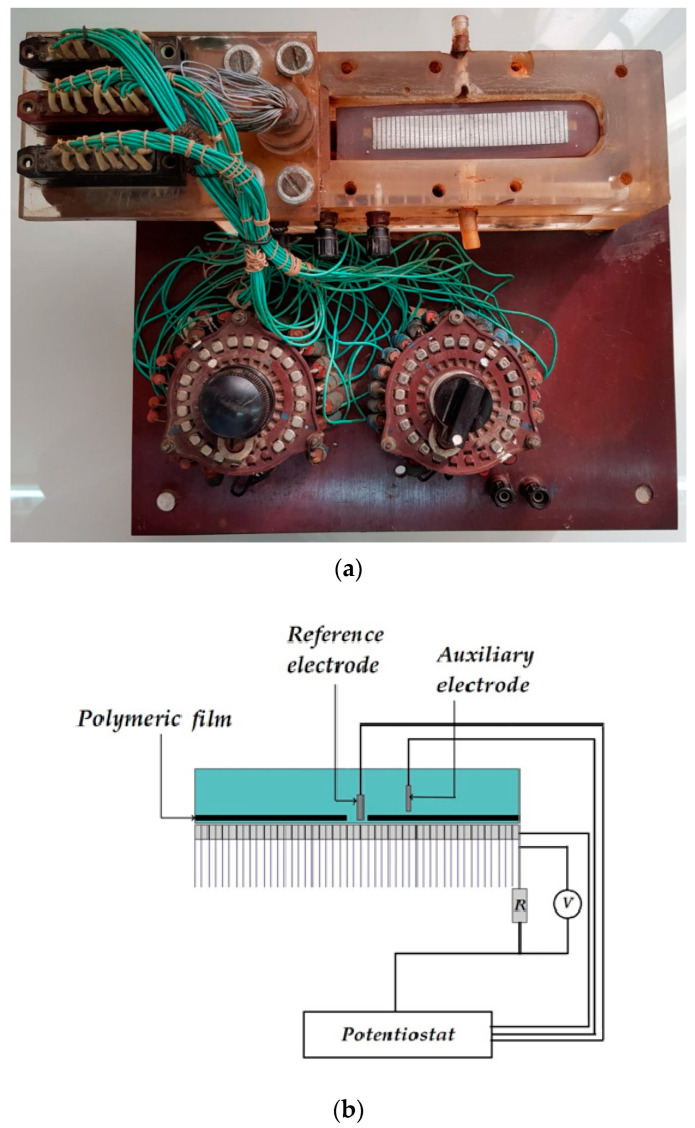

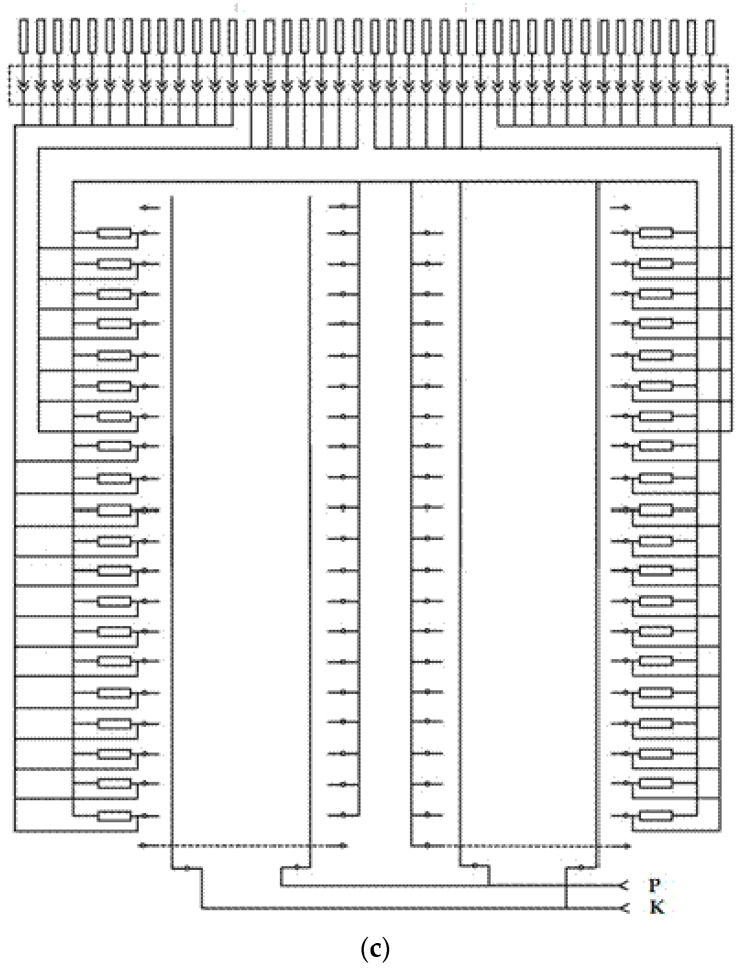
Cell for studying underfilm metal dissolution: (**a**) appearance of the cell; (**b**) schematic diagram of the cell for studying underfilm metal dissolution; (**c**) electrical circuit of the cell for studying underfilm metal dissolution.

To conduct experiments for studying the distribution of protective current and underfilm corrosion on steel, a cell shown in Figure 1, which is similar to that used in [26], was developed and manufactured. The working element is a set of 42 steel plates isolated from each other (each 15 × 2.3 mm in size and 0.34 cm^2^ in area). The total length of the metal part of the working element is about 10 cm. A polymer film with a defect simulating a peeled coating was mounted above the metal surface. Thе size of the gap between the metal and the polymer could be varied in the range of 0.5 mm to 2 cm. A polymeric film with a thickness of 100 μm and Polyken tape (manufactured by Polyken Technologies a division of Kendall Company, Westwood, MA, USA,) were used as coatings. This tape is part of the design of an insulating coating for underground gas and oil product pipelines, which comprises a butyl rubber primer (primer), a polymer tape, and a wrapping.

The defect in the coating was located above plates 20 and 21, i.e., approximately in the middle of the working assembly. The space between the polymer coating and the working electrode was filled with an electrolyte. A cathodic potential was supplied to the plates from a potentiostat to simulate the action of a cathodic station on a real pipeline operating under electrochemical protection (ECP). A Luggin capillary was placed near the surface of the metal under the coating near the defect. It was used to set and measure the potential of the steel under the coating. The potentials were measured relative to a silver-silver chloride reference electrode and recalculated to the scale of a copper sulfate reference electrode (c.s.e.), usually applied for field measurements of the potentials of underground metal structures [7,27,28]. All the potentials in this work are presented relative to the copper sulfate reference electrode. A platinum electrode was used as the auxiliary electrode for electrochemical studies. The current flowing from each plate of the working element was measured. To do so, the voltage drop across calibrated resistors connected to the plates (terminals P and K (Figure 1b,c)) was measured. The variation in the current with the distance from the defect in the polymer coating was recorded. Using such a cell made it possible to simulate the peeling of a polymer coating from the metal and map the metal surface to a coating peeling defect based on the current distribution. A sodium chloride solution was used as the working electrolyte, in which pitting corrosion of steel usually occurs.

**Figure 2:**
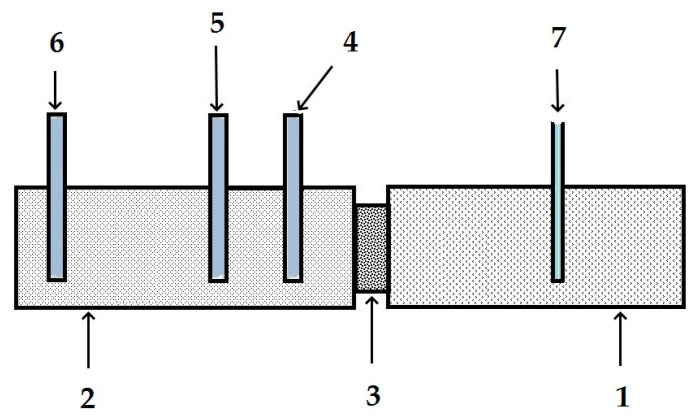
Diagram of a cell for studying oxygen diffusion through a polymer membrane. 1—cell compartment for oxygen input; 2—working cell compartment; 3—polymer membrane; 4—platinum electrode; 5—reference electrode; 6—auxiliary electrode; 7—oxygen input.

To determine the possibility of the cathodic reaction of oxygen reduction on the metal under a peeled insulating film at a distance from a defect (or in the case of a defect-free corrugation) at distances at which gas diffusion in a thin electrolyte layer is hindered (or not possible), oxygen diffusion through a polymer membrane was studied. For this purpose, a cell similar to that described in [29] was used, which makes it possible to measure the penetration rate of medium components (in this case, gas) through polymer membranes with different thicknesses. The cell is shown schematically in Figure 2. Oxygen was fed into one compartment of the cell (1) by purging the solution with an oxygen flow at a constant rate. Oxygen diffused through the polymer membrane (3), entered the working compartment of the cell (2), was deaerated with argon, and was reduced on the working platinum electrode (4).

The oxygen reduction current was measured, from which the permeability of oxygen and the diffusion coefficient of oxygen in membranes of various types were calculated. A cathodic potential of 0.500 V (Cu/CuSO_4_ reference electrode) was applied to the working electrode. The following buffer solution was used as the working electrolyte: 0.1 M Na_2_SO_4_ + 0.07 M Na_2_B_4_O_7_ + 0.15 M H_3_BO_3_ (pH = 8.7).

Profilometric measurements (i.e., determination of the surface profile: assessment of roughness and corrosion penetration depth [30]) were carried out using a TR-100 surface roughness tester (manufactured by TIME GROUP Inc., Beijing, China).

The influence of silanes on the electrochemical behavior of metals was studied using polarization methods. Anodic polarization curves have been recorded. A three-electrode cell with a silver chloride reference electrode was used. The measurements were carried out using a potentiostat PI-50-1 (manufactured in the Republic of Belarus) at fixed potentials or potentiodynamically with a potential sweep rate of 0.1, 0.2, and 1 mV/s. Experiments were carried out on disk electrodes made of carbon steel, grade St3, with an area of 3.14 cm^2^, disk (0.8 cm^2^), and cylindrical (12.14 cm^2^) aluminum electrodes (highly pure aluminum with Al content 999–99%), coated with a siloxane polymer coating and butyl rubber primer (steel) and epoxy resin (aluminum). When carrying out electrochemical studies, the following working solutions were used: 0.3 M Na_2_SO_4_, pH 6.4 (siloxane-coated steel); 0.1 M Na_2_SO_4_, pH 6.3 (steel with butyl rubber coating); 0.1 M NaCl, pH 6.2 (aluminum with epoxy resin); borate buffer solution (pH 6.5) with the addition of 0.001 M NaCl (steel coated with a bitumen-polymer coating). For electrochemical experiments, samples were selected that had the same polymer coating thickness and similar ohmic resistance. Anodic polarization curves of steel samples coated with silicone rubber in a solution of 0.3 M Na_2_SO_4_ in distilled water were obtained on rotating disk electrodes (S = 1.1 cm^2^, ω = 1010 rpm). The potential sweep rate was 60 mV/min. Steel disks pressed into an organic glass holder were prepared according to the method already described. We used a potentiostat PI-50-1 (manufactured in the Republic of Bedarus); potentials were measured relative to a silver chloride electrode аnd recalculated to the scale of a standard hydrogen electrode. Before applying the polymer coating, the sample was prepared as follows: the working surface of the sample was ground with fine-grained sandpaper, washed with distilled water, degreased with acetone, dried in a desiccator over phosphorus oxide, immersed in a 5–10% solution of an orgsanosilane in toluene (or water) for 10 min, and washed with dry toluene. All toluene was dried over sodium. After such modification, a polymer coating was applied to the working surface. Modification of polymeric coatings with organosilanes was carried out by introducing organosilane into the coating volume (to a concentration of 0.5–1.5 wt.%) before curing the coating. When studying the effect of organosilanes on the electrochemical behavior of steel coated with a bitumen-polymer coating (0.5–0.8 mm thick), anodic polarization curves were also recorded. A three-electrode cell with separated electrode spaces has been used; the potential was measured relative to the silver chloride reference electrode and calculated on the normal hydrogen scale. Measurements were carried out using potentiostat I PC-Pro (made in Russia) potentiostatically (at fixed potentials) or potentiodynamically (with a potential sweep rate of 0.1 mV/s). Three types of steel electrodes were used as samples: disk S = (0.8 cm^2^), cylindrical (S = 12.14 cm^2^), and rectangular (length 15 mm, width 12 mm, thickness 0.3 mm). The auxiliary reference electrode was a platinum plate with an area of 1 cm^2^ and a thickness of 1.2 mm.

Critical pitting potential *E_pit_* (or local depassivation potential of steel), i.e., the potential above which pitting dissolution of the metal occurs and stable pitting is formed, was determined from the anodic polarization curves by the break in the curve as the potential upon reaching which a sharp increase in current was observed. The criterion for the inhibitory activity of organosilane was the magnitude of the shift of *E_pit_* (Equation (1)) towards positive values:

(1) $$\Delta E_{pit}=E_{pit-mod}-E_{pit-bgd}$$

where *E_pit-mod_* and *E_pit-bgd_* are the values of the pitting potential in the presence (*E_pit-mod_*) and absence (background coating—*E_pit-bgd_*) of the modifying organosilane additives, respectively.

## 3. Results and Discussion

**Figure 3:**
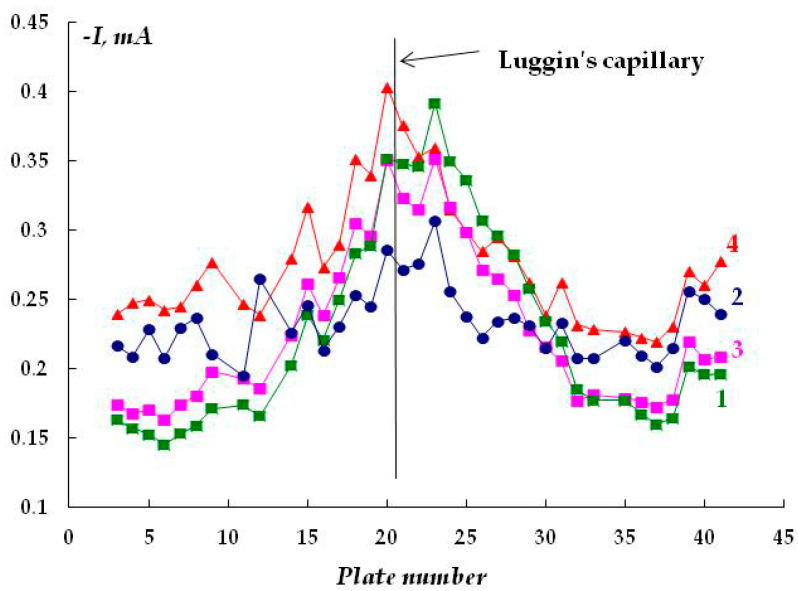
Dependence of the cathodic current on the concentration of chloride ions in the solution. Cell without a polymer coating. E = −1.2 V. Polarization time: 40 min. NaCl concentration, M: 1—0.15; 2—0.2; 3—0.25; 4—0.5.

The cathodic behavior of carbon steel without a polymer coating was studied. Figure 3 shows the plots of cathodic currents on the distance from the capillary tip of the reference electrode (Luggin capillary) in sodium chloride solutions at a cathodic potential of −1.2 V, which lies in the range of potential values recommended by regulatory documents [31,32,33] for the ECP of underground structures.

It can be seen in Figure 3 that the cathodic current is the largest on the plates located near the capillary tip of the Luggin reference electrode, and the current values decrease with the distance due to the contribution of the ohmic component of potential (ohmic drop [34,35,36,37]). Increasing the chloride concentration from 0.15 M to 0.5 M does not have a significant effect on the cathodic current variation (Figure 3). This is apparently due to the mutual compensation of two effects: on the one hand, an increase in salt concentration increases the conductivity of the electrolyte, but on the other, it reduces the solubility of oxygen. Changing the chemical composition of the electrolyte did not lead to a significant change in the electrochemical behavior of steel, either. Namely, replacing sodium chloride with sodium sulfate (at the same concentrations) yielded a similar distribution and similar cathodic current values.

**Figure 4:**
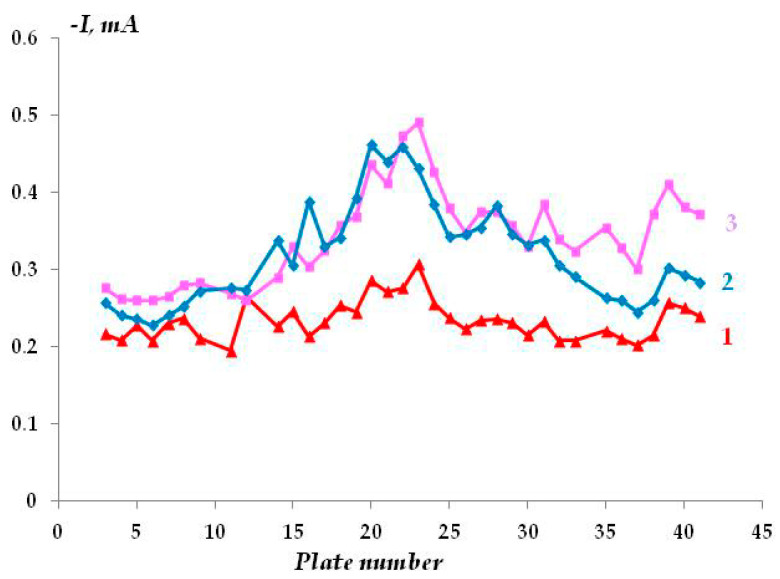
Dependence of the cathodic current on the time of polarization without a polymer coating in 2 M NaCl, E = −1.2 V. Polarization time: 1—40 min, 2—115 min, 3—231 min.

In a highly concentrated (2 M) solution, the effect of the air-oxide film on the steel surface on the distribution of protective current can be noticed. Initially, the ohmic potential drop is determined by the resistance of the oxide film, so the distribution of protective current is fairly uniform (Figure 4, curve 1). With the increasing time of cathodic polarization, the oxide film is reduced, and an increase in the cathodic current is observed. A normal distribution of the cathodic current appears, with a maximum on the plates located close to the tip of the Luggin capillary (Figure 4, curves 2 and 3). After 115 min of polarization, the current value remained approximately constant, with the exception of the current maximum near the tip of the Luggin capillary (Figure 4, curve 2). Thus, in the absence of an insulating coating, the current variation is predominantly affected by the ohmic potential drop determined by the resistance of the electrolyte solution and the distance from the Luggin capillary.

**Figure 5:**
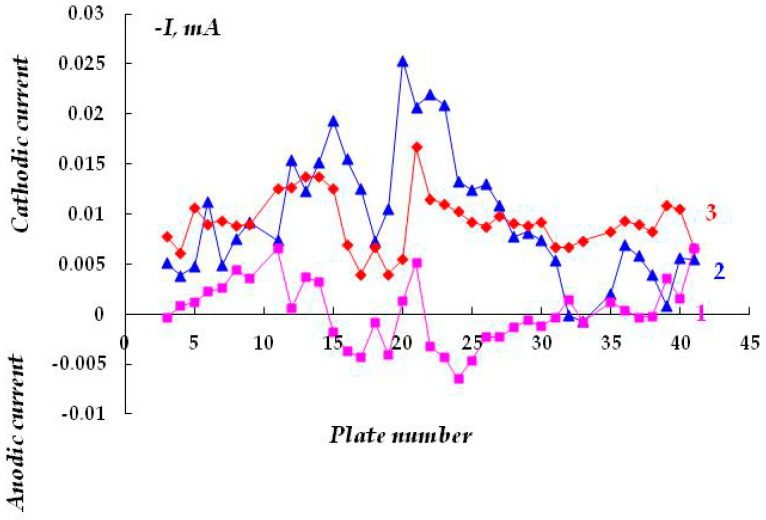
Effect of the cathodic polarization time at an under protection potential on the magnitude and sign of the current flowing from the metal. Polarization time: 1—20 min; 2—120 min; 3–160 min. The potential is E_pol_ = −0.640 V (c.s.e.) (E_cor_ = −0.638 V). The Luggin capillary was located near the 21st plate.

It can be assumed that in a surface area with a peeled coating, the metal is dissolved in the anodic zones, whose area is determined by the area of the cathodic and passive zones. It may be expected that in the case of reliable and long-term maintenance of the cathodic protection potential along the entire length of a metal under a peeled coating, the hazard of underfilm dissolution of the metal section is small; however, it is unlikely that such cases of uninterrupted operation of the ECP in the design mode are met in practice. As the ECP potential shifts to the anodic region and approaches the steel corrosion potential, or at short polarization times, the probability of the formation and localization of cathodic and anodic areas on the metal in places of coating defects increases. The corrosion rates in anodic areas can be high and result in the formation of cavities and pits. In Figure 5, curve 1, one can see that even in the absence of a coating, a change in the direction (reversal of polarity) of the current flowing from the metal plates from cathodic to anodic occurs, though the entire metal sample is cathodically polarized. This indicates the high probability of accelerated dissolution of the metal in such places on the surface. Accelerated dissolution of the metal under the peeled-off polymer coating in small anodic areas can lead to the formation of small cavities and pits on the metal surface. Such local defects can act as stress concentrators under conditions of simultaneous exposure of the structure to a corrosive environment and mechanical loads that occur on extended underground metal structures (for example, on main pipelines) and cause the initiation of corrosion cracks [38].

Increasing the polarization time from 20 to 120 min leads to a decrease in the area of the anodic areas of metal dissolution (Figure 5, cf. curves 1 and 2), while after polarization for 160 min, no polarity reversal was observed (no anodic current was recorded on any metal plate), and only cathodic current was recorded on the entire examined surface (Figure 5, curve 3). Thus, cathodic polarization provides suppression of metal dissolution, even though the potential is shifted cathodically just a little from the corrosion potential.

The current under a peeled polymer coating is affected by two main factors:

(a) the ohmic potential drop, whose effect is more significant the greater the distance from the defect to the Luggin capillary and the thinner the electrolyte layer between the polymer and the metal (i.e., the higher the resistance of the electrolyte layer);
(b) the oxygen concentration gradient in the underfilm electrolyte.

**Figure 6:**
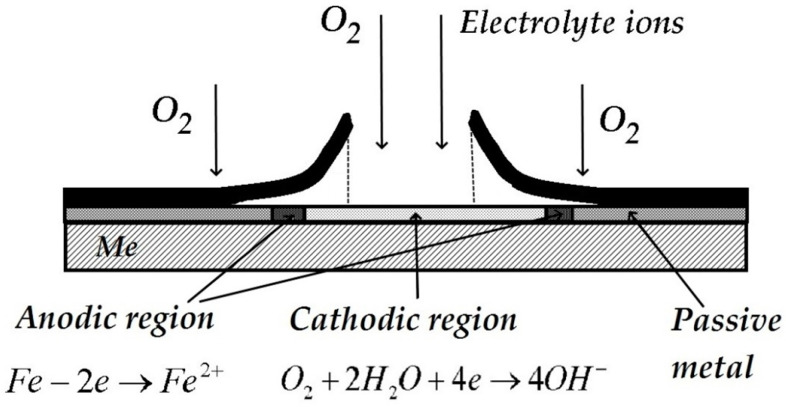
Metal-electrolyte interface in the presence of a polymer coating with a defect.

If the polymer coating peeled off from a sufficiently extended metal section (a few centimeters long), an oxygen concentration gradient emerged in the underfilm electrolyte. The diagram for this case is displayed in Figure 6. The most oxygen-enriched layer is the electrolyte layer, located directly under the through defect in the coating (region I, Figure 6). The oxygen concentration in the solution layer under the coating decreases with distance from the defect (region II, Figure 6). The limiting stage of the corrosion process in this case is the delivery of oxygen to the metal surface, and conditions for the existence of so-called “differential aeration pairs” arise under the peeled coating [39,40,41] (Figure 6).

**Figure 7:**
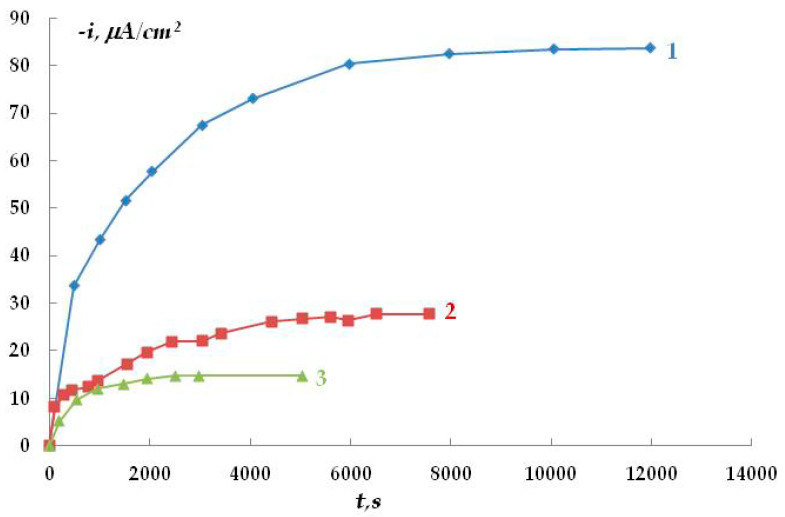
Variation in the rate of oxygen penetration through the polymer membrane over time. 1—I = 30 µA, E = −0.75 V; 2—I = 30 µA, E = −0.50 V; 3—I = 80 µA, E = −0.50 V.

The kinetics of oxygen penetration through polymer membranes were studied. The results are presented in Figure 7.

Based on the data obtained, it may be asserted that polymer membranes are permeable to oxygen. Oxygen diffusion depends on the type of polymer coating, potential, and temperature. As the potential shifts towards negative values (more negative than the corrosion potential), the rate of oxygen penetration through the polymer film increases. The diffusion coefficients for polymer coatings were calculated. Their values are k_PE_ = 0.21582 × 10^−8^ cm^2^/s and k_PU_ = 1.154 × 10^−8^ cm^2^/s for polyethylene and polyurethane, respectively. The values obtained are close in order of magnitude to the published diffusion coefficients in polymers [42]. The steady-state rate of oxygen penetration through a 1 mm thick polymer membrane was 1.06 µA/cm^2^, while the possible diffusion of oxygen through the polymer coating in air was 0.2 µA/cm^2^.

Thus, regardless of the state of the polymer coating, oxygen penetrates the metal surface. However, the maximum flow of oxygen apparently exists at places where there are through defects in the coating.

It can be expected that in places of free access to oxygen, namely near a defect in the polymer coating, the cathodic reaction (Equation (2)) will predominantly occur on the metal surface:

(2) $$O_{2}+2H_{2}O+4e\to 4OH^{-}$$

As the distance from the defect increases (in the areas to which the access of oxygen is hindered), anodic areas with an increased metal dissolution (Equation (3)) rate will emerge.

(3) $$Fe-2e\to Fe^{2+}$$

Thus, in the absence of an insulating coating, the change in current value will predominantly be affected by the ohmic potential drop determined by the resistance of the electrolyte solution and the distance from the Luggin capillary.

**Figure 8:**
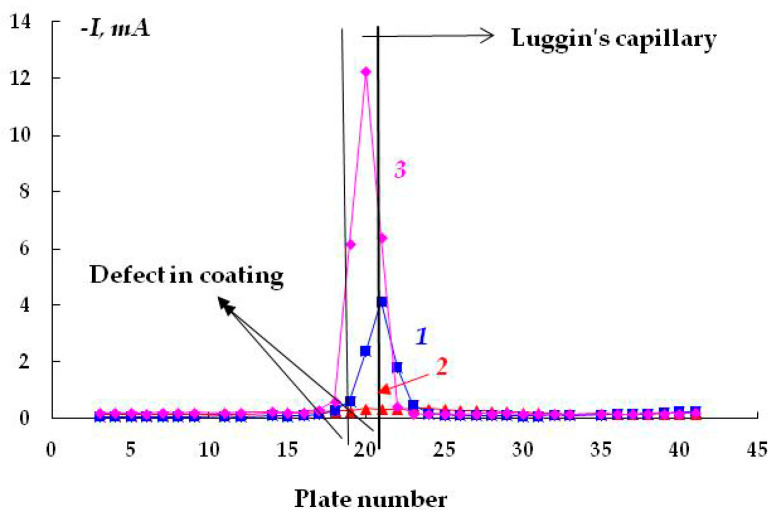
Cathodic currents flowing from the metal (plates made of carbon steel St3) with (1, 3) and without (2) the coating. Curve 3 corresponds to the presence of the coating; the metal is after 12 h of free corrosion. The gap between the coating and the metal is 1 mm; 0.15 M NaCl; E = −1.2 V.

The distribution of cathodic current on steel plates coated with a polymer film with a defect located above the 20th and 21st plates was studied. The studies were carried out in a 2 M NaCl solution at a potential of −1.2 V (Figure 7). The Luggin capillary of the reference electrode was placed near plate 21, i.e., directly under the defect in the insulation in the middle of the sample (Figure 1b). As it can be seen in Figure 8, the magnitude of the cathodic current is determined by the distance from the defect in the polymer coating. Similar to the case without a coating, the maximum current values were observed on the central plates. The maximum in the dependence of the current value on the distance from the defect (and from the Luggin capillary) became significantly narrower on displacement from the defect even to a small distance, which may be indicative of uneven cathodic protection of the metal under a peeled-off polymer. Apparently, only the metal area in the immediate vicinity of the defect in the insulation is provided with sufficient cathodic protection, and a significant drop in the protective current was observed even at a distance of less than 1 cm from the defect (Figure 8).

As the distance from the defect increases (at a distance of 10 mm with a gap thickness of 1 mm), a decrease in the current from 4.13 to 0.15 mA (Figure 8, curve 1) is observed, apparently due to the ohmic potential drop. The contribution of the ohmic component of the potential increases with a decrease in the thickness of the electrolyte layer due to an increase in the electrical resistance of the solution. In the experiments where cathodic polarization was switched off periodically (12 h of polarization followed by 12 h without polarization), visual observations showed the presence of steel corrosion under the coating over the entire surface of the sample. When a potential was applied after 12 h of corrosion, the value of the cathodic current at the defect site was significantly higher compared to the initial values. The current reached 12.5 mA (Figure 8, curve 3), which may be due to the reduction of iron oxides/hydroxides.

**Figure 9:**
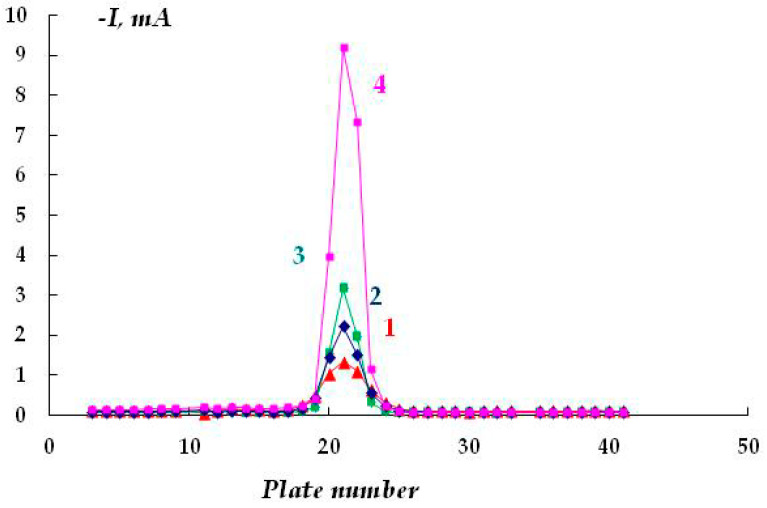
Dependence of the cathodic current on the distance between the coating with a defect and the metal surface. Gap between metal and coating: 1—4 mm; 2—3 mm; 3—2 mm; 4—0.5 mm. 0.15 M NaCl, E = −1.2 V.

The effect of the size of the gap between the polymer coating with a defect and the metal in 0.15 M NaCl, E = −1.2 V, was studied (Figure 9). It was shown that as the thickness of the underfilm electrolyte layer decreases from 4 mm to 0.5 mm, the cathodic current flowing from the plates located directly under the defect in the insulating coating increases from 1.32 to 9.19 mA.

As noted above, this effect is apparently due to the easier access of oxygen to the metal surface with a decrease in the thickness of the electrolyte layer and, hence, an increase in the efficiency of the cathodic process in accordance with Equation (4):

(4) $$i_{c}=kD\Delta C/\delta$$

where *D* is the oxygen diffusion coefficient, ΔC is the oxygen concentration gradient, δ is the thickness of the electrolyte layer, and *k* is a constant.

**Figure 10:**
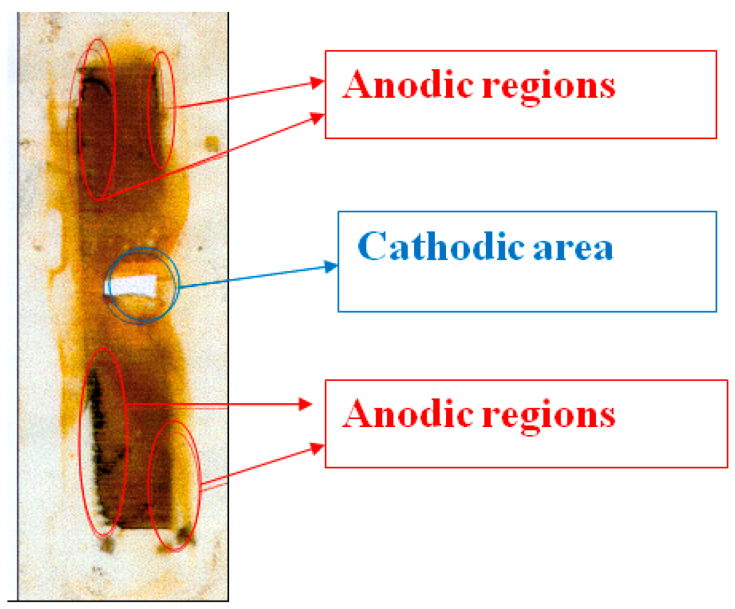
Replica (on polyethylene film) of a steel surface that corroded under a polymeric coating.

Figure 10 shows a replica (on polyethylene film) of a steel surface that has corroded under the insulation. Corrosion products accumulate on the metal surface due to periodic shutdowns of ECP. The entire surface is covered with an orange layer of rust that presumably consists of Fe_2_O_3_. However, it is possible to visually distinguish the cathodic and anodic areas (Figure 5; the corrosion products in the anodic areas are darker). Magnetite Fe_3_O_4_ is formed at the anodic sites due to a lack of oxygen during dissolution. It can be seen that the anodic areas are located at a distance from the defect in the insulation and are apparently due to the hindered access to oxygen. The cathodic zones, as one would expect, are located immediately next to the coating defect. When polarization was turned on (after 12 h of corrosion), cathodic current flowed from all steel plates (Figure 8), which indicates that the entire observed metal surface becomes cathodically protected when a cathodic potential is applied to the sample. A visual inspection of the metal surface after the experiment did not reveal pitting, i.e., local damage caused by depassivation of the surface (damage to the passive oxide film). The metal dissolved uniformly at each anodic site, though at different rates in different areas.

The metal dissolution rate under the polymer coating and, accordingly, the depth of damage are determined by the potential. A profilometric study showed that, the depth of damage in the anodic areas was naturally larger than on the rest of the metal surface and varied within 0.18–0.36 mm/year, while the mean corrosion rate over the entire surface was 0.11 mm/year.

Pits and cavities on the metal surface under peeled coatings, which are often observed under field conditions, appear to be the result of the localization of anodic areas. Moreover, depending on the conditions and characteristics of operation, the area of these sections can be very small compared to the entire area of the metal under the peeled coating. Visual inspection showed that the ratio of the areas of anodic and cathodic sections can vary from 1:10 to 1:100. During long-term operation, the corrosion penetration depth can be significant (the formation of pits and cavities). If such corrosion zones are localized to form local defects with diameters up to 1–10 mm, they can initiate the formation and growth of stress-corrosion cracks [4,38].

**Figure 11:**
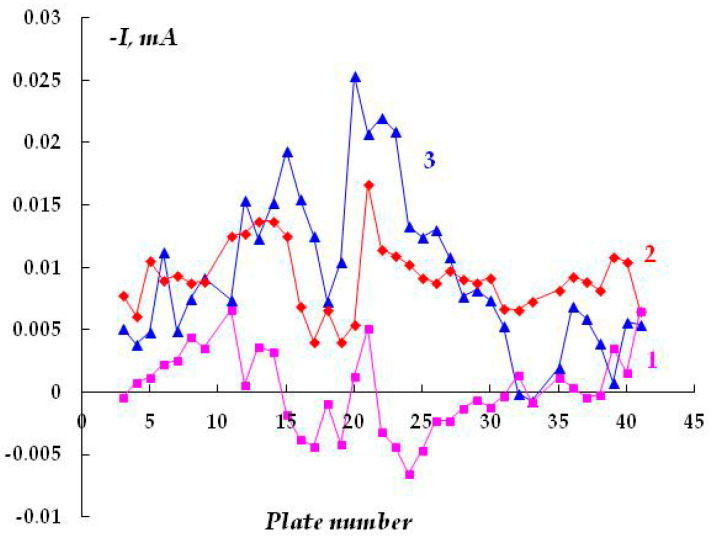
Time variation of the cathodic current under a coating with a defect in 0.15 M NaCl. E = −0.75 V (E_cor_ = −0.647 V). Polarization time, minutes: 1—20; 2—250; 3—cathodic polarization for 12 h followed by 60 h of corrosion.

The corrosion behavior of steel under a peeled coating was studied at potentials smaller (in absolute value) than the ECP potentials and close to the steel corrosion potentials (E = −0.75 V). It has been shown (Figure 11) that anodic areas are present on the metal (polarity reversal) at distances of 4 cm from the defect (Figure 11, curve 1). After some time (250 min), all plates turned out to be cathodically polarized (curve 2). Long-term polarization, 12 h followed by switching off, also led to a current polarity reversal in small areas of the surface. Nevertheless, it has been shown that anodic areas exist under an insulating coating during cathodic protection of the metal. It should be stressed that the presence of anodic zones on the surface of the metal under the coating results in increased metal dissolution, despite the overall cathodic polarization of the entire structure. If the area of the anodic sections is small, one can expect a transition from uniform to much more hazardous local dissolution of the metal. Periodic or accidental switching off of cathodic polarization would lead to the persistence or even aggravation of these effects, along with general steel corrosion. An increase in the gap between the insulating coating and the metal led to a decrease in the area of anodic sections. At the maximum (in our case) gap size of 8 mm, anodic sections were not detected, neither by instruments nor visually. In this case, the resulting dependence of current vs. the distance from the defect is similar to that described above without a polymer coating.

Thus, the separation of the metal surface into anodic and cathodic regions is due to the ohmic potential drop in the underfilm electrolyte and the oxygen concentration gradient. The specific feature of the electrochemical behavior of steel under an insulating film is related to the drop in current and potential under the insulating film, the inefficient action of cathodic protection with the distance from a defect in the insulation, and, as a consequence, the localization of anodic and cathodic areas on the surface with an increased rate of dissolution at the anodic areas. In the presence of a thick conductive layer of underfilm solution, the cathodic protection system operates satisfactorily, and dissolution of the metal under the coating is unlikely unless the protection is switched off. However, even in these cases, the dissolution will be uniform and is unlikely to pose a danger during short ECP shutdowns.

**Figure 12:**
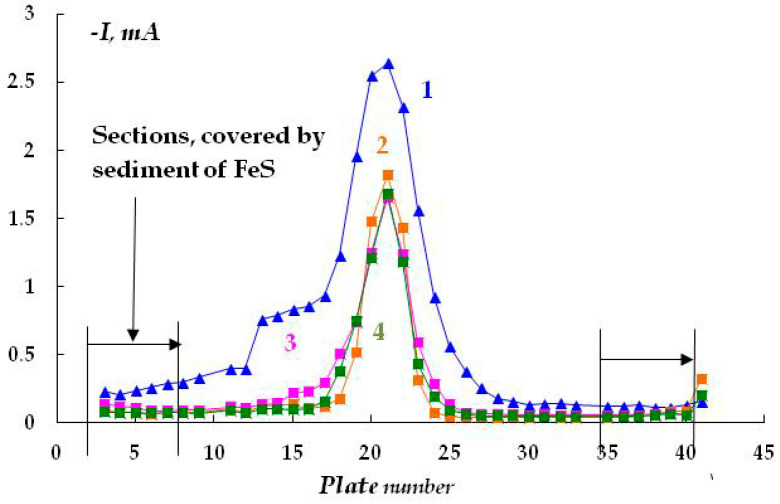
Time variation of the cathodic current under a coating with a defect in 0.15 M NaCl with the addition of 0.001 M H_2_S, E = −1.2 V. Polarization time, minutes: 1—90; 2—165; 3—235; 4—345.

As concerns the initiation of stress corrosion cracking, the effect of such important corrosive components of natural soils as hydrogen sulfide and carbon dioxide (or hydrogen sulfide- and carbonate-containing compounds) on underfilm corrosion was examined. We studied the effect of adding hydrogen sulfide and carbonate-containing compounds to the background underfilm working electrolyte (0.15 M NaCl, pH 4.5 with the addition of 0.1 M Na_2_CO_3_) on the underfilm dissolution of the metal. The smallest height of the gap between the coating and the metal was used. The addition of hydrogen sulfide (concentration 0.001 M) to the electrolyte at a potential of −1.1 V did not lead to a significant change in the nature of cathodic current distribution (compare Figure 8 and Figure 12). The cathodic currents, both near the defect in the insulation and at a distance from it, were even slightly smaller than in the background solution (0.15 M NaCl). Moreover, in the presence of hydrogen sulfide in the electrolyte and in the pure sodium chloride solution, the cathodic current flowing from the metal near the defect in the insulation was found to decrease with time. Moreover, in the presence of hydrogen sulfide, black coloration of the metal was observed on the plates located on both sides at a distance of 3.5 cm from the coating defect after 8 h of the experiment. It was apparently caused by the accumulation of a deposit of poorly soluble sulfide on the surface. This indicates that dissolution of the metal occurs under cathodic protection, i.e., anodic processes occur on these plates, leading to the emergence of iron ions (in accordance with Equation (3)) and their subsequent interaction with sulfide ions, as demonstrated in Equation (5):

(5) $$Fe^{2+}+H_{2}S\to FeS+2H^{+}$$

and accumulation of a black deposit on the metal surface.

**Figure 13:**
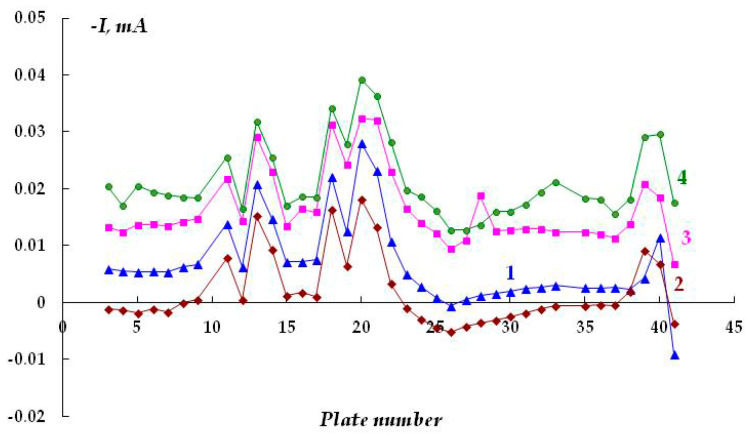
Time variation of cathodic current under a coating with a defect in 0.15 M NaCl with the addition of 0.001 M H_2_S, E = −0.75 V (E_sor_ = −0.73 V). Polarization time, minutes: 1—15; 2—75; 3—150; 4—260.

In the presence of sulfide-containing compounds (0.1 M NaCl + 0.001 M Na_2_S) in the underfilm electrolyte, a small potential shift (from the corrosion potential) in the cathodic direction (E_cat_ = −0.75; E_cor_ = −0.73 V) led to the formation of anodic sections with a larger area than in the background electrolyte without the addition of sodium sulfide (Figure 13). However, although the anodic currents are small and close to those in the background solution, the results obtained indicate that the dissolution of steel is activated by hydrogen sulfide and sulfide-containing compounds. For example, even after cathodic polarization for 75 min, reversal of current polarity (from cathodic to anodic) was observed on 23 plates (out of 45), i.e., under the coating in the presence of hydrogen sulfide-containing compounds. Even with cathodic polarization, the area of the anodic sections (where the metal dissolves) exceeded half of the metal surface.

**Figure 14:**
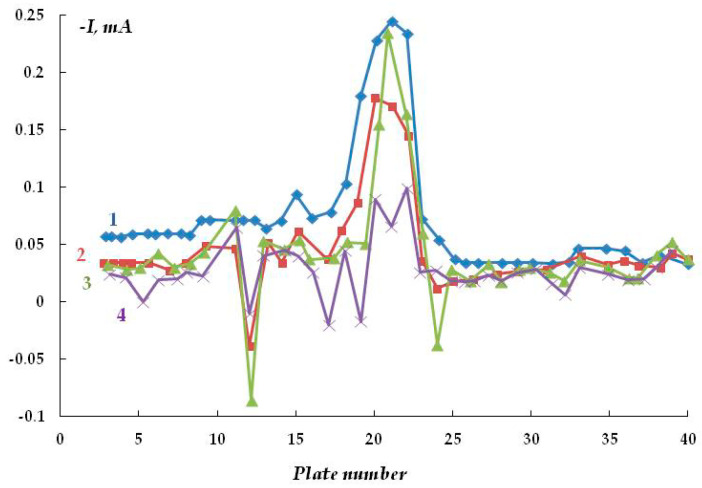
Time variation of cathodic current under a coating with a defect in 0.15 M NaCl with the addition of 0.003 M H_2_S, E = −1.2 V. Polarization time, minutes: 1—15; 2—95; 3—1500; 4—2940.

**Figure 15:**
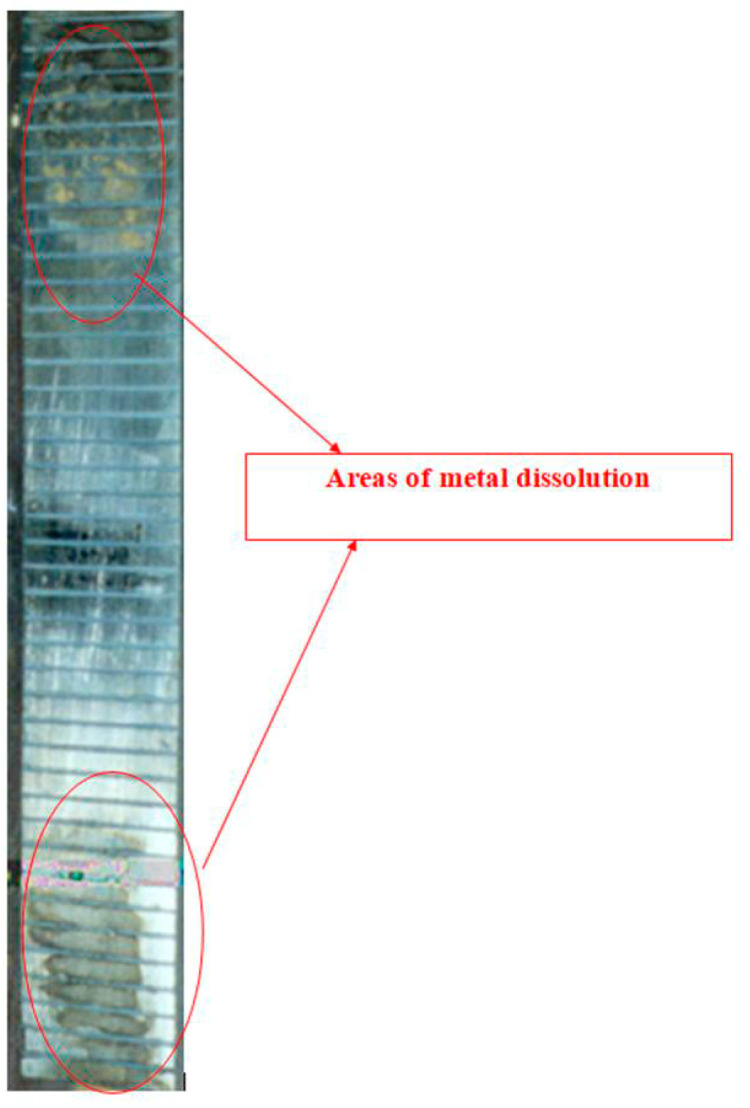
Steel surface after 3 days of cathodic polarization at E = −1.2 V (after removal of corrosion products from the surface) in 0.15 M NaCl electrolyte with addition of 0.003 M H_2_S; pH 4.5. Surface areas where the metal is dissolved are highlighted in red.

An increase in the sodium sulfide concentration from 0.001 to 0.003 M leads to the occurrence of anodic currents (Figure 14) even at a high cathodic potential of −1.2 V that meets the criteria for cathodic corrosion protection of low-alloy steels, i.e., more negative than −0.85 V relative to the sulfate reference electrode [7,27,32,33]. Moreover, after continuous cathodic polarization at a potential of −1.2 V for 3 days, pronounced and visually identifiable areas of anodic dissolution of the metal were observed (Figure 13 and Figure 15).

**Figure 16:**
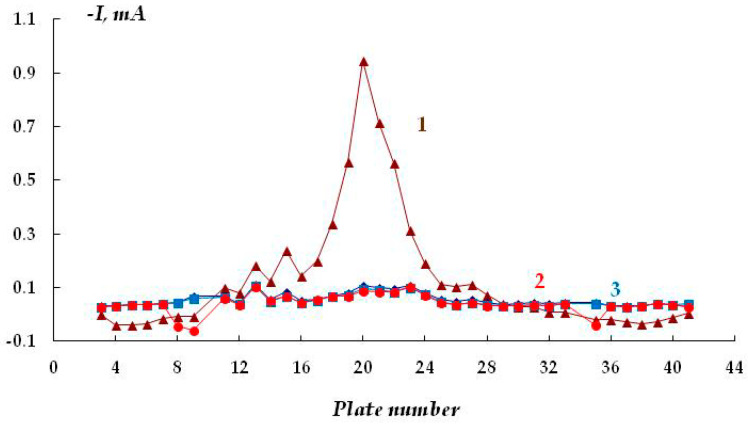
Time variation of cathodic current under a coating with a defect in the electrolyte: 0.15 M NaCl + 0.1 M Na_2_CO_3_, pH 4.5. E = −1.2 V. Polarization time in minutes: 1—0; 2—720 min of polarization and 720 min of corrosion; 3—180.

In addition to hydrogen sulfide, the localization of anodic sites on the metal surface under the coating is caused by the presence of CO_2_ in the solution (background solution: 0.15 M NaCl, pH 4.5). For example, anodic areas were observed upon periodic switching the cathodic polarization on/off (12 h of polarization, 12 h of free corrosion, total test time 6 days) (Figure 16).

Corrosion in the presence of carbonate-containing compounds can involve the following reactions on the steel surface (Equations (6) and (7)):

(6) $$Fe^{2+}+2H_{2}CO_{3}\to Fe{\left(HCO_{3}\right)}_{2}+2H^{+}$$

(7) $$Fe^{2+}+H_{2}CO_{3}\to FeHCO_{3}+2H^{+}$$

**Figure 17:**
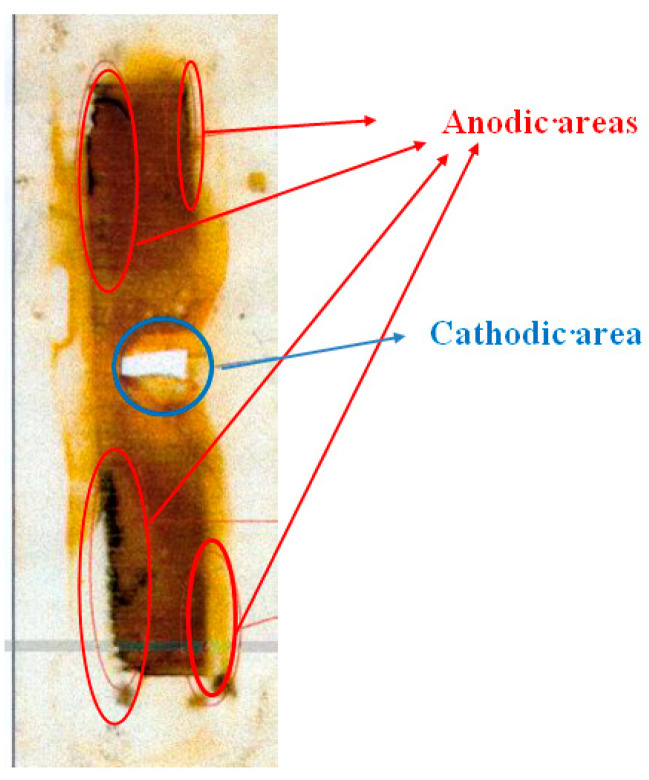
Replica (on polyethylene film) of steel surface after corrosion under insulation in the presence of 0.1 M H_2_CO_3_.

A replica of this case is presented in Figure 17.

Figure 17 shows that the anodic surface areas are located at a distance from the insulation defect. The cathodic region is located near the defect.

**Figure 18:**
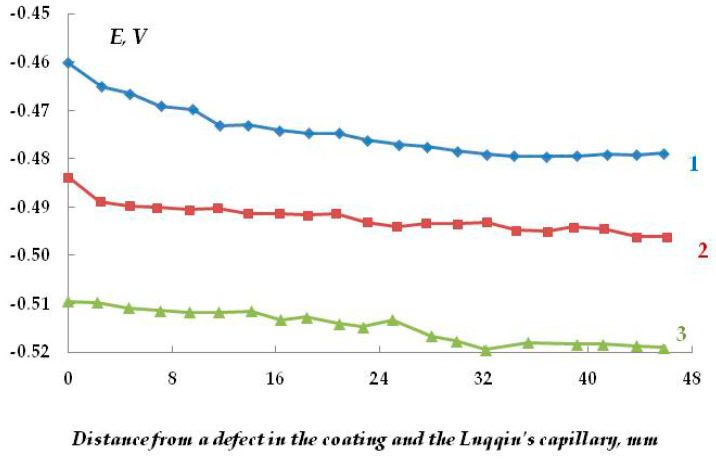
Potential distribution over the steel surface under a coating with a defect in 0.15 M NaCl with the addition of 0.001 M H_2_S. Polarization time: 1—80 min; 2—27.5 h; 3—52 h.

Measurements of the free corrosion potential of steel under the coating in the presence of hydrogen sulfide in the electrolyte showed that this potential decreased after cathodic polarization; (moreover, the decrease was higher the longer the preliminary polarization was (Figure 18)), which was not observed in the background electrolyte. This confirms the conclusion about the activation of corrosion processes in the presence of hydrogen sulfide. Measuring the change in corrosion potential with distance from the defect also showed that the potential shifted in the negative direction by 15–20 mV, which may be indicative of a decrease in the O_2_ flow (at a distance of 5–10 cm from the defect in the insulation), which also confirms the possible occurrence of more active metal areas (in terms of corrosion) as the distance from the defect increases.

Thus, the presence of even small amounts of hydrogen sulfide and/or carbon dioxide in the underfilm electrolyte leads to dissolution activation in localized anodic areas under the insulating coating.

To simulate the environments that actually exist on the pipeline route, the corrosion-electrochemical behavior of steel was studied in an artificial electrolyte with a composition matching the solution in the delaminated insulation on a section of the real main gas pipeline. Analysis of the solution from the corrugated insulation gave the following composition: 0.92 g/L Na_2_CO_3_; 0.1 g/L KCl; 0.1 g/L NaCl, 0.1 g/L CaCl_2_, 0.1 g/L MgSO_4_·7H_2_O, and 0.1 g/L H_2_S (iron ions present in the solution were not analyzed).

**Figure 19:**
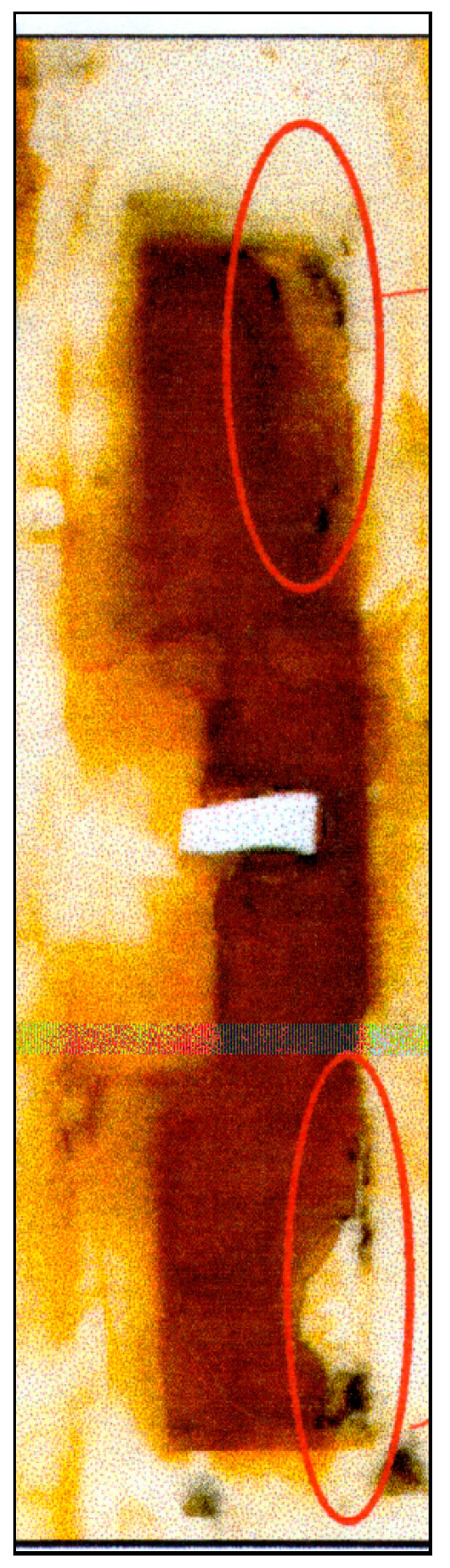
Replica of steel corroded under the insulation under cyclic cathodic loading in an electrolyte from the corrugated insulation of an operating main gas pipeline. Red circles indicate areas of the surface where accelerated anodic dissolution of the metal occurs.

**Figure 20:**
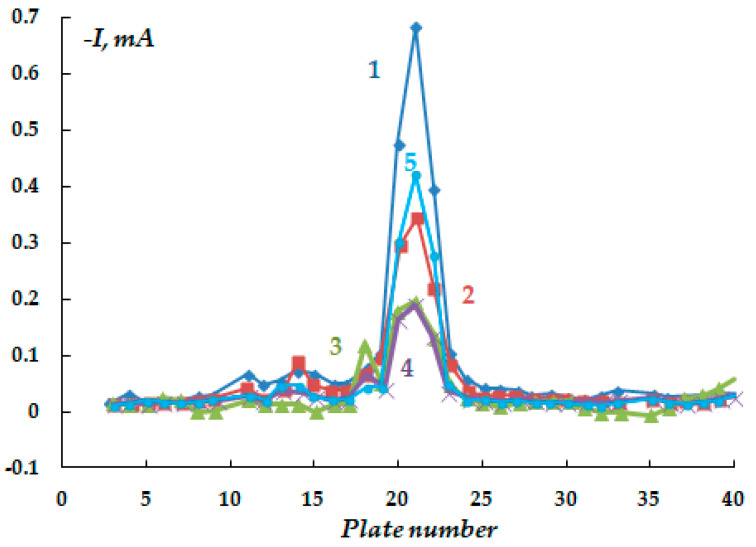
Time variation of cathodic current under a coating with a defect in a solution from a corrugated coating on a real pipeline, E = −1.2 V. Polarization time, minutes: 1—10; 2—60; 3—150; 4—320; 5—1440.

It has been found that despite the low content of corrosive ions in the electrolyte (total ionic strength 0.017), the formation of anodic areas on the metal is observed on the surface even at a cathodic potential of the entire sample (Figure 19), although the magnitude of the cathodic currents flowing from the defect in the insulation is smaller in this case than in more concentrated solutions (Figure 20).

Thus, electrochemical studies carried out on a setup simulating a defect in pipeline insulation showed that in the presence of a peeled polymer coating (corrugation), only the metal located in the immediate vicinity of the defect is provided with cathodic protection. In the presence of an electrolyte under a peeled polymer coating, local anodic areas can emerge on the metal even at a small distance (5–10 cm) from the defect in the insulation. Anodic dissolution of the metal in these areas occurs both in the case of free corrosion (when the ECP is turned off) and when a cathodic protective potential is applied to the structure. The presence of hydrogen sulfide or carbon dioxide in the underfilm electrolyte activates the metal dissolution, which is manifested as an increase in the area of local anodic spots compared to the background electrolyte (at potentials close to the stationary one) and in their occurrence at high cathodic potentials (without turning off the ECP).

Since stress-corrosion damage is the most hazardous type of metal destruction under the conditions of the pipeline route, and according to a number of concepts, the crack in the metal appears due to local corrosion damage (pitting) [4], we studied the effect of hydrogen sulfide and carbon dioxide on the pitting dissolution of steel. As noted above regarding the study of the underfilm metal dissolution, no visible pitting was observed on the surface; however, this does not imply that pitting on steel is not possible under certain conditions.

**Figure 21:**
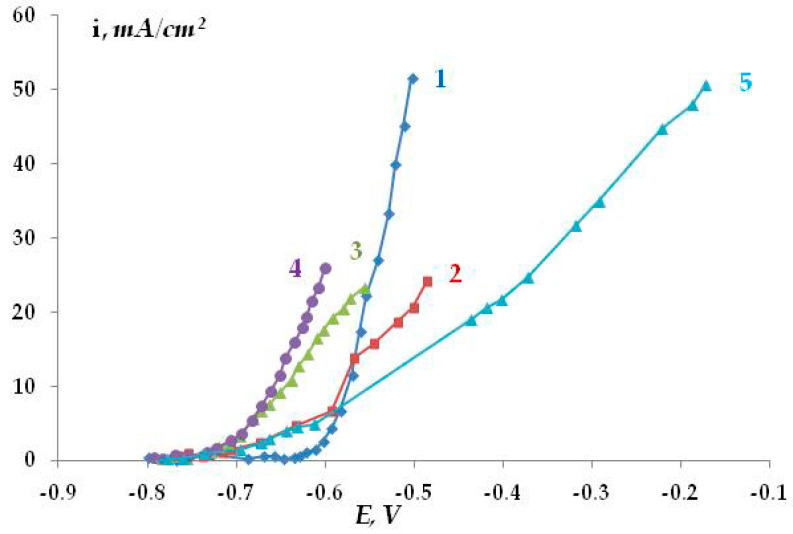
Anodic potentiodynamic curves. Time variation of cathodic current under a coating with a defect in 0.1 М Na_2_SO_4_, рН 6.2, E = −1.2 V. 1—steel in a background solution without additives of sulfide and/or carbonate. Hydrogen sulfide addition: 2, 3, 4. Carbon dioxide addition: 5.

A not-too-corrosive (in terms of pitting сorrosion) 0.1 M sodium sulfate solution was chosen as the background electrolyte. Figure 21 shows the anodic polarization potentiodynamic curves of steel in this solution. A sharp break in curve 1 at a potential of −0.61 V, followed by a significant increase in the anodic current, indicates the occurrence and development of pitting on steel in this solution. Visual inspection of the samples confirmed the presence of pitting on the metal surface. Small pits with a diameter of 0.1 mm and a depth of 0.1–0.2 mm were observed. The surface density of pits after recording the anodic curve was 80–90 per cm^2^. We believe that it is unlikely that the presence of such corrosion sites can initiate the growth of a corrosion crack. The addition of hydrogen sulfide (curves 2–4) or carbon dioxide (curve 5) to the background electrolyte led to a break in the curve at higher cathodic potentials, which indicates some activation of the pitting process. However, the flatter shape of the curves compared to the background solution may indicate that the development of the pits that have already formed is inhibited. The salt deposit formed on the metal (which necessarily occurs during anodic polarization in the presence of hydrogen sulfide or carbon dioxide) can screen the surface, so the true anodic current values cannot be measured. Visual inspection of the samples after the removal of the precipitated dissolution products showed the presence of pits on the metal surface. Their geometric dimensions and quantity were almost the same as in the pure background solution without additives. The data obtained show that hydrogen sulfide and carbon dioxide have some ability to activate pitting corrosion in steel, but the degree of this activation is insignificant.

**Figure 22:**
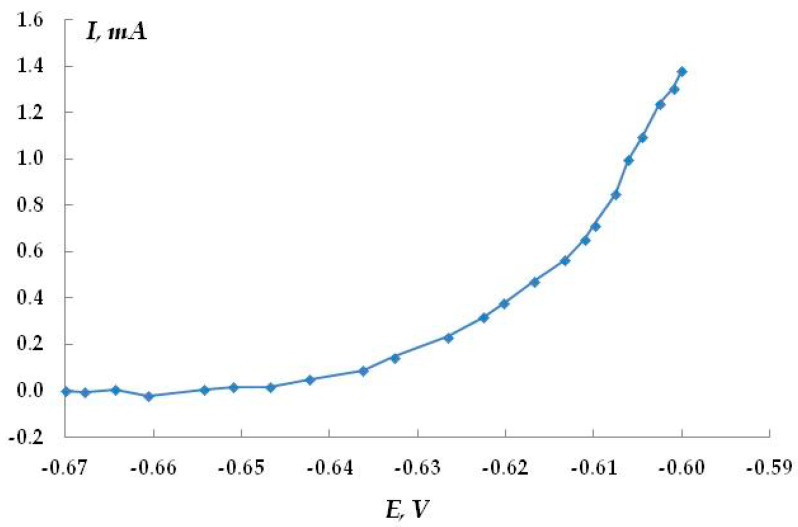
Anodic potentiostatic curve of steel in 0.1 M Na_2_SO_4_, pH 4.5.

**Figure 23:**
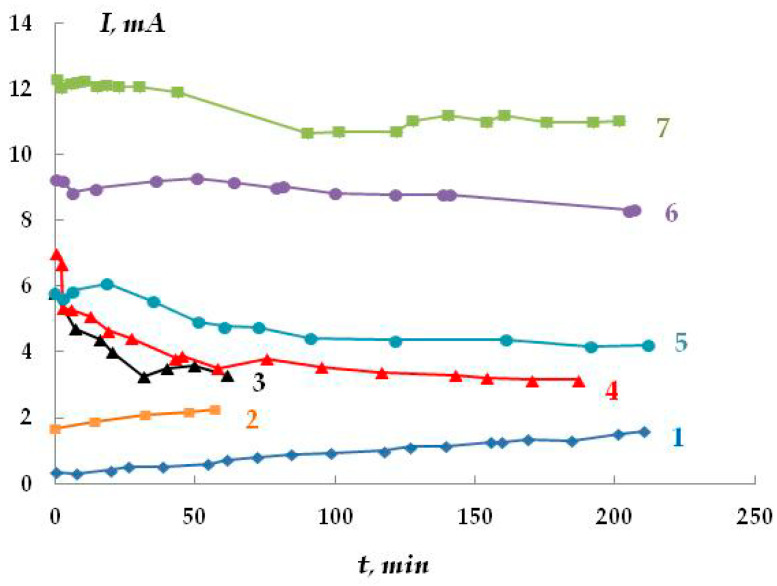
Kinetics of anodic current variation at E = −0.65 V in 0.1 M Na_2_SO_4_ (1) and in the presence of 0.0005 M H_2_S (2); 0.1 M H_2_CO_3_ (3); 0.01 M H_2_CO_3_ (4); 0.001 M H_2_CO_3_ (5); 0.01 M H_2_CO_3_ and 0.0005 M H_2_S (6); 0.1 M H_2_CO_3_ and 0.0005 M H_2_S (7).

Anodic potentiostatic polarization makes it possible to determine the potential at which the pitting process begins more reliably. In the case of steel in 0.1 M Na_2_SO_4_ (pH 4.5), the pitting potential is E = −0.625 V (Figure 22). To determine the effect of hydrogen sulfide and carbon dioxide present in the electrolyte on the anodic behavior of steel, we studied the kinetics of pit development upon a small shift from the pitting potential (E = −0.65 V). The kinetic curves are presented in Figure 23. It has been shown that in the background electrolyte, the anodic current increases with polarization time, which indicates the activation of the anodic dissolution of steel. However, the small slope of the curve indicates that the process develops insignificantly over time at this potential. The presence of hydrogen sulfide in the solution (concentration 0.0005 M or 17 mg/L) leads to a slight increase in the rate of anodic dissolution (slope of the curve 0.016 mA/min) compared to the background electrolyte (slope of the curve 0.0058 mA/min). In contrast, the addition of carbon dioxide to the solution decreases the efficiency of the anodic process (the slope of the curve is 0.034 mA/min). The effect of the addition of hydrogen sulfide and carbon dioxide to a 0.15 M NaCl solution was studied (Figure 23). Chloride ions are more corrosive than sulfate ions in terms of the pitting corrosion of metals. Despite this, the data obtained are similar to the results for the sulfate-containing electrolyte. The anodic dissolution of steel in the presence of hydrogen sulfide or carbon dioxide occurred at rates exceeding those of the anodic dissolution of steel in the pure chloride-containing electrolyte. The time variation of the stationary potential in the presence of hydrogen sulfide in the solution may also be indicative of possible pitting corrosion. However, the data obtained are insufficient for making an unambiguous statement about the activating or inhibitory effect of hydrogen sulfide and carbon dioxide on the pitting corrosion of steel. In the case of dissolution in the presence of H_2_S and/or CO_2_, a deposit was observed on the surface, which can distort the measured current values. However, if hydrogen sulfide and carbon dioxide activate the development of pitting dissolution, it occurs to a very small extent and is clearly insufficient to explain the activation of stress corrosion cracking by these compounds.

**Figure 24:**
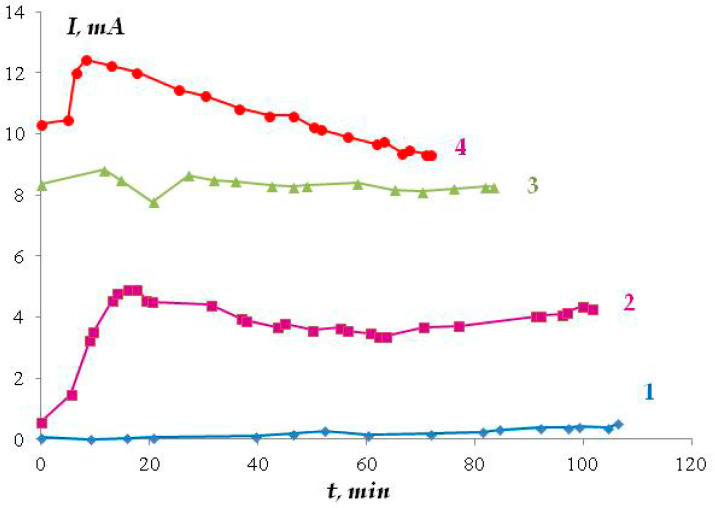
Time dependence of the anodic current in 0.15 M NaCl pH 4.5; E = −0.3 V in 0.15 M NaCl (1) and with addition of 0.1 M H_2_CO_3_ (2); 0.1 M H_2_CO_3_ and 0.0005 M H_2_S (3); 0.0005 M H_2_S (4).

We believe that the stress corrosion activating effect of hydrogen sulfide and carbon dioxide is associated not only with the development of pitting corrosion but also with the activation of (uniform) anodic dissolution. For example, in the presence of H_2_S and CO_2_, the stationary potential of steel in sulfate- and chloride-containing solutions shifted by 40–50 mV in the negative direction, which may indicate the activation of the corrosion (anodic) process. Moreover, and this is very important, the addition of hydrogen sulfide or carbon dioxide to the solution led to an increase in the metal dissolution current by more than an order of magnitude (Figure 23 and Figure 24). For example, at a potential of −0.65 V in a sulfate-containing electrolyte, the anodic current was 0.5 mA, while, for example, the addition of 0.0005 M H_2_S increases the anodic current to 3–5 mA. It can be seen that even very small amounts of the additives result in such an increase in the dissolution current. With increasing concentrations of the additives, the values of anodic currents increase (Figure 24). A similar effect was observed in both sulfate- and chloride-containing background electrolytes. Apparently, hydrogen sulfide and carbon dioxide act as anodic active compounds, increasing the efficiency of anodic steel dissolution. Moreover, in some cases (Figure 24, curves 6 and 7), we observed a synergistic effect, i.e., the combined presence of hydrogen sulfide and carbon dioxide in the solution led to an increase in the anodic current, and the current value exceeded those measured in the presence of each of these compounds separately at the same concentration. Similar effects have been documented in the literature for these compounds in other environments. The interpretation of this synergistic effect requires additional studies. However, it is of importance that the presence of hydrogen sulfide, carbon dioxide, or their derivatives in the electrolyte leads to a significant increase in the rate of metal dissolution in the anodic areas.

**Figure 25:**
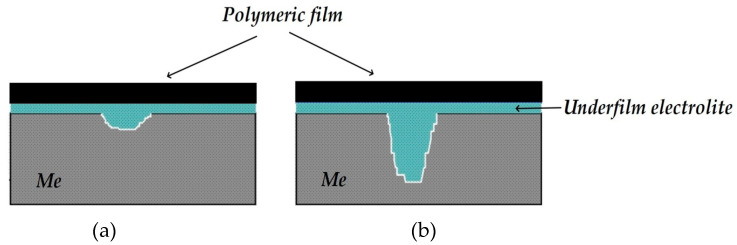
Scheme of corrosion damage on anodic areas of the metal under the insulating coating: (**a**)—in the absence; (**b**)—in the presence of hydrogen sulfide and/or carbon dioxide.

We suggest the following mechanism for the underfilm dissolution of the metal and the effect of hydrogen sulfide and carbon dioxide on the corrosion-based destruction of the metal (including stress corrosion). When a defect occurs in the insulating coating, the polymer peels off from the metal over time. The soil electrolyte penetrates into the gap thus formed between the insulation and the pipe surface. Only the area of the metal directly under the defect in the insulation is completely provided with cathodic protection. Even at a cathodic potential, and especially when the cathodic potential is turned off temporarily or permanently, anodic areas are localized on the metal surface under the peeled insulation, their area being significantly smaller than the total surface of the metal under the peeled insulation. In these anodic areas, enhanced dissolution of the metal occurs, and, over time, corrosion pits may appear on the surface (Figure 25a). Although the metal from the anodic areas dissolves uniformly in general (though pits may appear under certain conditions), the general state of the metal corresponds to local dissolution due to the large difference in the anodic and cathodic areas. Moreover, microlocalization of the anodic sections with geometric dimensions up to several millimeters in diameter can apparently occur under certain conditions (for example, see Figure 25). Localization of anodic areas under the polymer coating will occur in all cases where the electrolyte penetrates the metal surface. However, although such localization results in uneven dissolution of the metal manifested as the presence of corrosion damage in anodic areas, it may be insufficient to initiate a corrosion crack (upon generation of mechanical stresses) due to low dissolution rates and small depths of corrosion penetration.

The penetration of carbon dioxide and/or hydrogen sulfide into the underfilm electrolyte (mainly due to the metabolism of sulfate-reducing soil bacteria) results in an increase, by more than an order of magnitude, in the rate of dissolution of the metal from anodic zones with approximately the same area, which can be manifested in the formation of defects (cavities) more than 10 times deeper (Figure 25b). This is especially hazardous if microlocalization of anodic areas occurs. The microcavities that arise in this case may act as initiators of a corrosion crack, which is apparently what happens in real life. If the conditions of pitting dissolution appear, the microlocalization process is aggravated, and the probability of crack initiation apparently increases. However, this is not a prerequisite because uniform dissolution from the anodic micro-areas can also cause a crack to occur.

Thus, the specific feature of the action of hydrogen sulfide and carbon dioxide is that they have an anodic-activating effect, as a result of which the rate of metal dissolution increases significantly, leading to the emergence of deeper local corrosion cavities and initiators of stress corrosion cracks.

Let us now consider the prospects for inhibiting the underfilm dissolution of a metal. Given that nearly all metal products (buildings, structures, and vehicles) used in various industries and in various environments (atmosphere, soil, seawater, river water), are protected by coatings (polymer, paint, varnish, etc.) in operation, developing ways to prevent metal corrosion under a polymer coating is a very important scientific and engineering problem. 

It is known [11] that under conditions where a coating is saturated with an aqueous electrolyte and oxygen, the corrosion rate of the metal in the initial period is low and is controlled by adhesion at the metal-polymer interface. During operation, defects often occur in the coating, or its adhesion is lost locally, which can significantly accelerate electrochemical processes and underfilm corrosion of the metal. Historically, it was assumed that polymer coatings protected the substrate from corrosion by acting as a barrier to water, oxygen, and ions [19]. However, detailed studies have shown that oxygen and water saturate the coating relatively quickly and cannot be the factors determining the metal corrosion rate [19]. It was found in [13,43,44] that ionic resistance is a key factor in the stability of a polymer coating. A relationship between the ionic resistance of a coating and its protective ability was reported [13,29]. Ionic resistance was also singled out in more recent studies [45,46] as an important (and even critical) characteristic of coatings in terms of their anticorrosion resistance. However, the concept that the ionic resistance of coatings is the most significant factor determining the anticorrosion efficiency of coatings is also criticized [47]. The presence of microgalvanic couples under a coating leads to its rapid failure. It is believed [19] that coating failure usually begins with local defects, which can be the result of coating errors, chemical heterogeneity of the coating, etc. Near these defects, coating adhesion is lost (so-called peeling caused by corrosion), a corrosive electrolyte penetrates through the metal-polymer interface, and the process of underfilm corrosion begins. On the other hand, the importance of adhesion of the polymer coating to the base, and especially preservation of adhesion under wet conditions (the so-called “wet adhesion”), is noted. Moreover, it has been asserted that adhesion is the key factor determining the overall service life of coatings [48,49].

In addition, due to the complex interactions of the polymer with the metal base, the role of adhesion and maintenance of the protective properties of coatings is far from being fully understood to date. In addition, studies presented in the literature find no quantitative connection between adhesion and corrosion protection, but many believe in a link [50]. In the course of studies conducted over several decades, various points of view have emerged, varying from the mandatory presence of phase water films on the metal surface (i.e., disruption of the adhesive bond) for the onset of underfilm corrosion [51,52] to the consideration of adhesion and corrosion as two independent phenomena [53,54,55]. The adhesion of the coating to the substrate usually decreases during the operation of a metal with a polymer coating in wet or liquid environments. Beginning from the initial moment, adhesive strength decreases, first significantly and then slightly. It was hypothesized that the decrease in adhesion is due to the migration of water molecules along the polymer-metal interface or through the polymer to the metal, especially in defective coating zones [56,57]. When a coating is applied, gas microcavities remain between the polymer and the steel base due to the microroughness of the steel surface. Upon contact with the liquid phase, they are filled with water that can migrate along the phase boundary [58]. In this way, a mono- or polymolecular layer of adsorbed water is formed at the metal-polymer interface.

The relationship between the protective properties of polymer coatings, adhesion, and moisture permeability was studied in [59,60,61]. It was found that polymer protective coatings, which feature significant permeability but retain high adhesion, provided good protection. Due to the adhesive interaction force field, the diffusion of water through the coating slows down compared to the free film [60]. This phenomenon is explained by the partial blocking of the active metal centers prone to water adsorption by adhesive bonds.

Based on the above considerations, it can be expected that strengthening the interface interactions at the metal-polymer interface would favor the prevention of both the initiation and occurrence of underfilm corrosion of the metal. It is known [52] that surface preparation is a necessary technological operation in the application of coatings and the formation of adhesive bonding. Technically competent standardized surface preparation can ensure a reproducible surface [62] (and therefore a reproducible initial adhesive strength) and the long-term performance of the adhesive contact. Moreover, it was stated in some publications that “Surface preparation is the key to bond durability” [63].

In addition, one of the purposes of surface preparation may be to introduce an additional intermediate layer into a system by creating it on the substrate surface before its contact with the adhesive. It is believed [62] that the formation of such a layer is one of the most promising ways to increase interface interactions at the metal-polymer interface. To form such surface layers, compounds called “adhesion promoters” [64] or “coupling agents” [65] are used. Of the organic compounds used for these purposes, such as organotitanates, organozirconates, benzotriazoles, etc. [66], the class of organosilicon adhesion promoters, namely organosilanes (or silanes), whose chemical formula is RnSi(OR’)_4−n_, is distinguished for its unique properties [66,67,68,69,70,71]. The specific feature of these compounds is manifested in the versatility of their promoting effect on substrates and polymers of various types.

Moreover, preliminary modification (pretreatment) of metal surfaces with organosilanes may not only improve the adhesion of the polymer coating to the metal [66,67,68,69,70,71], but also inhibit metal corrosion [67,68,69]. In some publications, the option to modify polymer coatings by incorporating organosilanes into their bulk is discussed. For example, modification of high-density polyethylene enhances the adhesion and hydrophilicity of the polymer [72]. We have shown in our previous study that organosilanes incorporated into the bulk of bitumen-polymer coatings for underground gas pipelines can inhibit corrosion cracking [73]. Taking the adhesion-promoting and corrosion-inhibiting properties of organosilanes into account, it can be assumed that the formation of an intermediate organosilane-based organosilicon layer at the metal-polymer interface would help prevent the underfilm dissolution of the metal. Therefore, we carried out a study on the effect of organosilanes on the electrochemical properties of the metal in the presence of a polymer coating.

The effect of organosilanes on the electrochemical behavior of siloxane-coated carbon steel has been studied. We used a siloxane coating for the insulation of underground pipelines that consists of a siloxane primer (VIKSINT primer) and a siloxane tape applied on top (LETSAR-LPT). The primer used in this study is a linear siloxane elastomer (8):

(8) $$OH-\begin{array}{c} CH_{3}\\ |\\ Si\\ |\\ CH_{3} \end{array}-(O-\begin{array}{c} CH_{3}\\ |\\ Si\\ |\\ CH_{3} \end{array}-O{)}_{n}-\begin{array}{c} CH_{3}\\ |\\ Si\\ |\\ CH_{3} \end{array}-OH,n=5000-10000$$

**Figure 26:**
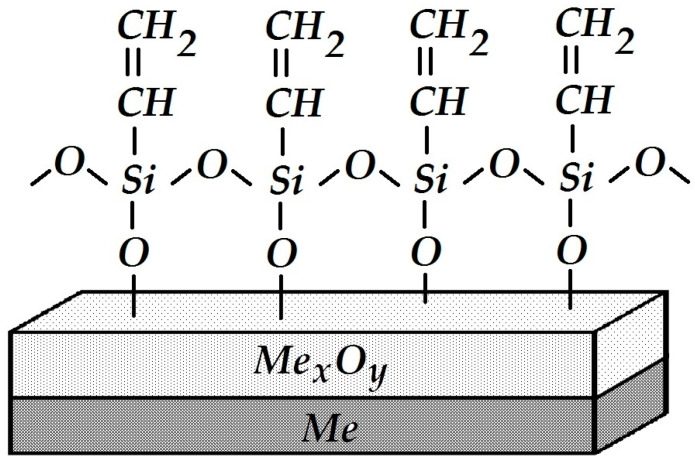
Schematic structure of the interphase boundary after modification of the steel surface with a trialkoxysilane.

It is cured by the addition of a tetraethoxysilane-based hardener, Si(OC_2_H_5_)_4_, due to cross-linking of the starting and ending silanol (≡Si–OH) groups; as a result, the length and mass of linear polymer chains increase, i.e., the polymer is cured. After polymerization (polycondensation), the elastomer molecules contain a small amount of polar hydroxyl groups that can form physicochemical bonds with the metal surface. One may assume that hydrogen bonds ≡Si–OH....HO–Me or covalent bonds ≡Si–O–Me are formed between the primer and the hydroxylated metal surface. The low resistance of hydrogen bonds to hydrolysis and their relatively small number explain the reduced water resistance and adhesion of such siloxane coatings. It can be hypothesized that the formation of a surface siloxane polymer (Figure 26) bound to the metal by covalent bonds with ≡Si–O–Me and serving as an intermediate sublayer for the application of a siloxane primer would facilitate adhesion and improve the anti-corrosion characteristics of the coating on steel.

The effect of organosilanes on the adhesion of a siloxane primer to steel was studied. If the steel surface is not modified, the adhesive strength amounts to A = 0.5 kg per cm of width. Modification of the steel surface with vinyltriethoxysilane (CH_2_=CH–Si(OC_2_H_5_)_3_ (VTES)) increased the adhesive strength more than two fold, up to A = 1.4 kg per cm of width. Thus, it was found that modification of the carbon steel surface with triethoxysilane results in enhanced adhesion at the metal-polymer interface. The effect of the nature of the organic radical R in the organosilane molecule on the adhesion of a siloxane coating to steel was examined. For example, if γ-aminopropyltriethoxysilane (NH_2_–(CH_2_)_3_–Si–(OC_2_H_5_)_3_ (APS)) containing a polar amino group in the organic radical was used, a slight decrease in adhesion of the coating to the metal was observed (A = 0.3 kg per cm of width). The use of epoxy-containing γ-glycidoxypropyltriethoxysilane (GPS—(9))

(9) $$H_{2}\begin{array}{c} /\\ C \end{array}\begin{array}{c} O\\ - \end{array}\begin{array}{c} \backslash \\ C \end{array}H-CH_{2}-O-{\left(CH_{2}\right)}_{3}Si{\left(OC_{2}H_{5}\right)}_{3}$$

trimethylsilylacetodecanetriethoxysilane (TADTES—(10))

(10) $${\left(CH_{3}\right)}_{3}-Si-O-C(O)-{\left(CH_{2}\right)}_{10}Si{\left(OC_{2}H_{5}\right)}_{3}$$

and, as noted above, surface treatment with vinylsilane (VTES) increased the adhesion strength compared to the unmodified metal, for which A = 0.5 kg per cm of width: A = 1.0, 1.2, and 1.4 kg, respectively.

The nature of coating separation from the surface of the metal modified with organosilanes was cohesive in all cases (i.e., destruction occurred due to both separation of the coating from the metal and destruction of bonds “within” the coating layer), whereas separation from the unmodified surface was always adhesive (i.e., 100% of the polymeric primer after tearing off remained on the tape, whereas the metal surface was free of coating residues).

The metal-polymer interface was strengthened. To do so, amino groups were grafted onto the coating by introducing 3 wt.% APS into the polymer bulk before curing. A silane containing an epoxy group in the organic radical (GPS) was applied to the steel surface.

**Figure 27:**
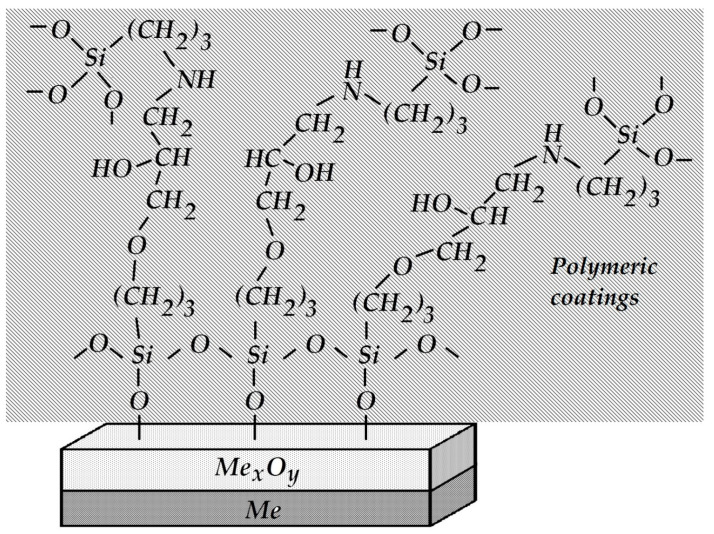
Schematic structure of the interphase boundary of the modified system consisting of GPS-modified steel and an APS-modified coating.

It is known that primary amines, in this case the NH_2_ group of aminosilane, are initiators of epoxy ring opening and addition at the ring opening position. Testing the adhesive strength of such a modified system demonstrated 100% cohesive peeling (the adhesive strength value was A = 1.0 kg per cm of width). In this case, the strength of coating adhesion to the metal was higher than the intrinsic strength of the siloxane elastomer itself, and the measured peel-off force did not correspond to the true adhesive strength at the metal-coating interface. The enhanced strength of the adhesive contact observed in this case is due to the mutual diffusion of the silane surface layer and the components of the polymer coating, the interaction between amino and epoxy groups, and the formation of the so-called “interpenetrating networks” [74]. A schematic structure of the interphase boundary is shown in Figure 27.

**Figure 28:**
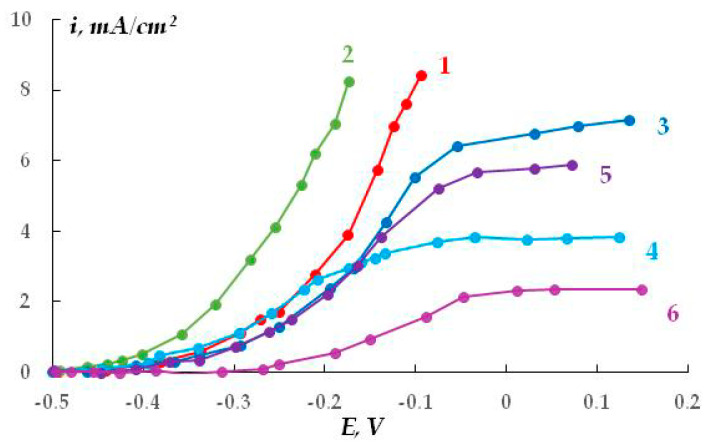
Anodic polarization curves of silicone-coated steel. Modification of the steel surface: 1, 2—without modification; 3, 5—GPS; 4, 6—TADTES. Coating modification: 1, 3, 4—none; 2, 5, 6—APS. 0.3 M Na_2_SO_4_, pH 6.2.

Electrochemical experiments were performed with samples that had the same thickness of the siloxane coating and similar ohmic resistances. Anodic polarization curves were recorded. The results are presented in Figure 28 and in Table 1.

The anodic polarization curves (Figure 28, Table 1) show that modification of the metal surface or coating with organosilanes results (with the exception of coating modification with aminosilane) in inhibition of the anodic dissolution of the coated steel (underfilm dissolution of steel). The greatest resistance to anodic dissolution of steel (at a potential E = E_cor_ + 500 mV) is exhibited by the coatings with the strongest adhesion, where the steel surface is modified with silanes and amino groups are grafted onto the siloxane coating (primer) (Table 1). The smallest polarization resistance was observed in the case of the siloxane elastomer modified with γ-aminopropyltriethoxysilane on the unmodified surface.

**Figure 29:**
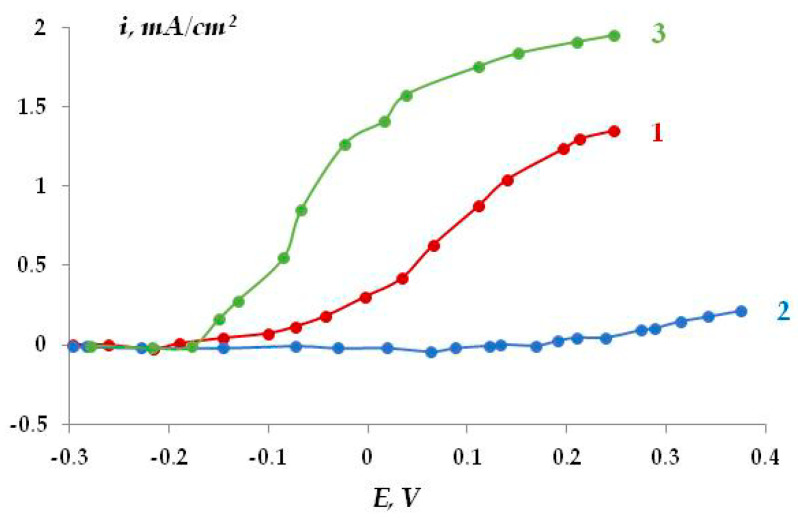
Anodic polarization curves of St3 with a primer based on butyl rubber: 1—without surface modification; 2—surface modification with VTCS; 3—addition of silane to the primer (10% VTCS in the primer). 0.1 M Na_2_SO_4_, pH 6.3.

In addition, steel samples coated with a butyl rubber coating (primer) were studied using electrochemical methods. Potentiodynamic polarization anodic curves of сarbon steel (grade St3) modified with vinyltrichlorosilane (VTCS) with subsequent application of a primer based on butyl rubber showed (Figure 29) that the highest polarization resistance to anodic dissolution of steel was found in electrodes that underwent preliminary modification of the steel surface with silane followed by application of a coating (Figure 29, curve 2). Incorporation of silane into the polymer matrix (Figure 29, curve 3) led to activation of the anodic process under the coating compared to the unmodified surface. This is apparently a result of the deterioration of the protective properties of the polymer due to the interaction of silane with the reactive groups in the coating, a decrease in the amount of reactive groups, and, as a consequence, a decrease in the probability of their binding to the metal [75].

Analysis of the experimental results shows that organosilanes inhibit the anodic dissolution of carbon steel under the polymer film. However, incorporation of silane into the polymer matrix did not enhance the protective effect, either in siloxane or polybutadiene polymer coatings.

Electrochemical studies of aluminum with a surface modified by APS were carried out in 0.1 M NaCl. For these studies, coatings of epoxy resin (grade ED-20) about 10 μm thick were formed on aluminum disk electrodes, unmodified or treated in aqueous solutions of APS (0.1 M). Before the measurements, coated electrodes were kept for 2 h in a 0.1 M NaCl solution, which ensured penetration of the electrolyte to the metal surface and a constant corrosion potential.

**Figure 30:**
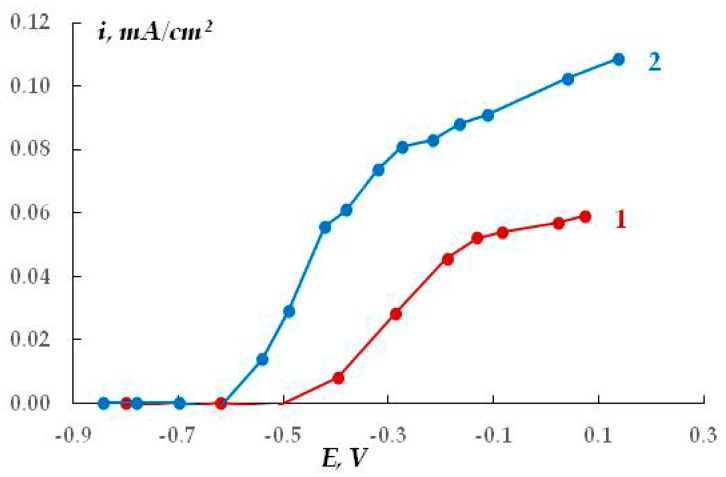
Anodic polarization curves of aluminum with a polymer coating (ED-20 epoxy resin): 1—without modification; 2—after modification with APS. 0.1 M NaCl, pH 6.0.

The resulting polarization curves are presented in Figure 30. The figure shows that, regardless of the surface pretreatment, application of the coating results in a significant shift in the critical pitting potential (E_pt_) in an anodic direction compared to the unpainted electrodes. The increase in E_pt_ indicates that local dissolution of the metal is inhibited. However, when aminosilane was used, a decrease in E_pt_ was observed due to the presence of amino groups on the surface. Moreover, E_pt_ decreased both in the presence of the coating (Figure 29) and without it [76].

Aside from the siloxane, butyl rubber, and epoxy coatings, steel samples with a bitumen-polymer coating were studied electrochemically. We used a bitumen-polymer coating (BP) of the Dekom brand (Delan, Moscow, Russia) widely applied for insulating underground gas pipelines. It consists of a bitumen-polymer primer, reinforced mastic, and polymer tape.

**Figure 31:**
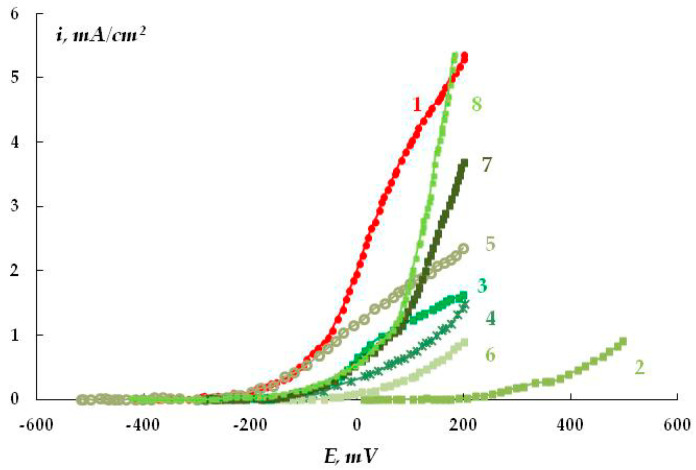
Anodic polarization curves of steel: 1—unmodified bitumen-polymer primer (BP) and BP modified with additives: 2—2% ODA; 3—1% VTES; 4—1% AGM; 5—1% APS + 1% VTES; 6—2% ODA + 1% VTES; 7—2% ODA + 1% APS; 8—2% ODA + 1% VTES + 1% APS. The primer layer thickness is 0.2 mm. Borate buffer solution (pH 6.5) with the addition of 0.001 M NaCl. Potential sweep rate: 0.1 mV/s.

The anodic polarization curves of metal samples whose surface was pre-treated with bitumen-polymer primers of various compositions (thickness 0.5–0.8 mm) are presented in Figure 31. The results show that the presence of an unmodified coating on the metal surface (without the addition of organosilanes) leads to passivation of the steel sample; no peak of the active-passive transition of the metal (Figure 31, curve 1) is observed, i.e., neither inhibition of uniform metal dissolution nor its passivation occur.

The rate of metal dissolution from pits, which is characterized by the slope of the anodic curve after reaching E_pt_, remains nearly unchanged for coated and uncoated samples (Table 2) and amounts to 0.013 and 0.014 (A·cm^2^)/V for coated and untreated steel, respectively. To modify the coating, we used not only organosilanes but also a popular corrosion inhibitor, octadecylamine C_18_H_37_NH_2_ (ODA) [77], since it is known [78] that the inhibitory ability of both organosilanes and organic corrosion inhibitors increases significantly when they are used as mixtures. The addition of modifiers (ODA, organosilanes, and their mixtures) into the bitumen-polymer primer leads to both a shift in E_pt_ in an anodic direction (Figure 31, curves 2–8) and a decrease in the rate of metal pitting dissolution (Table 2). This indicates that modifiers inhibit both the emergence of pitting and the rate of its development. The slopes of regions of anodic curves in the potential range more positive than E_pt_ are presented in Table 2. It can be seen that modification of the bitumen-polymer primer with organosilane-based formulations leads to inhibition of underfilm local dissolution of the metal. The greatest efficiency was observed when the coating was modified with a mixture of 2% ODA and 1% VS.

Thus, electrochemical studies have shown that organosilanes inhibit the dissolution of metal under polymer coatings.

## 4. Conclusions

1. The distribution of the protective current under a peeled polymer coating was studied, and a sharp decrease in the value of the protective current was shown to occur at a distance of 1–3 cm from the edge of the defect with a gap between the metal and the coating of 1–6 mm.
2. Localization of corrosion under the peeled polymer coating was found to occur. This effect is significantly enhanced by the presence of carbon dioxide or hydrogen sulfide in the corrosive environment. The ratio of the area of regions with accelerated corrosion to the total area of the metal can range from 1 to 100.
3. The presence of anodic areas under cathodic polarization of a metal with a peeled coating has been observed. The current flowing from the anodic areas increases approximately tenfold in the presence of carbon dioxide or hydrogen sulfide.
4. An activating effect of carbon dioxide and hydrogen sulfide on the anodic dissolution of steel was observed. A synergistic effect of these gases on the local corrosion of steel was discovered.
5. The mechanism that drives the formation of local corrosion damages in steel under a peeled insulating coating, which can serve as nuclei of corrosion cracks under a certain level of mechanical stress, was identified.
6. Ways for preventing the underfilm dissolution of metals were considered, and a method for inhibiting the dissolution of a metal under a peeled coating was suggested. The method involves the formation of an intermediate organosilicon layer at the metal-polymer interface after surface pretreatment with organosilanes.

## Figures and Tables

**Table 1:** Adhesive strength A kg per cm of width and anodic current I_a_ at E = E_cor_ + 500 mV on steel 3 with a siloxane primer, depending on steel modification and the primer (coating).

Modification		А*,* kg*/*cm	I_an_, mА
Steel	Primer		
No (Figure 28, curve 1)	No	0.5	1.00
GPS (Figure 28, curve 3)	No	1.0	0.72
ТАDТES (Figure 28, curve 4)	No	1.2	0.42
No (Figure 28, curve 2)	АPS	0.2	3.20
ВТES	No	1.4	0.80
ТАDТES (Figure 28, curve 6)	АPS	1.0	0.27

**Table 2:** Values of pitting potentials (E*_pit_*) and slopes of regions of anodic polarization curves (in the potential range more positive than E*_pit_*) of St3 carbon steel coated with a bitumen-polymer primer (BP).

Сoating Composition	E*_pit_* (s.h.e.), mV	tg а, mА·cm^2^/V
Steel without coating	−250	0.014
Steel + BP + 2% ОDА	−70	0.009
Steel + BP + 1% VS	−140	0.004
Steel + BP + 1% АPS	−110	0.006
Steel + BP + 1% VS + 1% АPS	−250	0.005
Steel + BP + 2% ОDА + 1% VS	−40	0.003
Steel + BP + 2% ОDА + 1% АPS	−170	0.008
Steel + BP + 2% ОDА +1% VS + 1%АPS	−190	0.012
Steel coated with unmodified BP	−250	0.013

## Data Availability

Data are contained within the article.
